# Dietary Perfluorohexanoic Acid (PFHxA) Exposures in Juvenile Zebrafish Produce Subtle Behavioral Effects across Generations

**DOI:** 10.3390/toxics10070372

**Published:** 2022-07-04

**Authors:** Yvonne Rericha, Lisa Truong, Connor Leong, Dunping Cao, Jennifer A. Field, Robyn L. Tanguay

**Affiliations:** 1 Department of Environmental and Molecular Toxicology, College of Agricultural Sciences, Oregon State University, Corvallis, OR 97331, USA; yvonne.rericha@oregonstate.edu (Y.R.); lisa.truong@oregonstate.edu (L.T.); connor.leong@oregonstate.edu (C.L.); jennifer.field@oregonstate.edu (J.A.F.); 2 Sinnhuber Aquatic Research Laboratory, Oregon State University, Corvallis, OR 97333, USA; 3 Department of Chemistry, College of Science, Oregon State University, Corvallis, OR 97331, USA; dunping.cao@oregonstate.edu

**Keywords:** PFAS, short-chain, toxicity, fecundity, multigenerational

## Abstract

Ubiquitous anthropogenic contaminants of concern, per- and polyfluoroalkyl substances (PFAS) are frequently detected in the environment and human populations around the world. Diet is a predominate route of human exposure, and PFAS are frequently measured in food. Manufacturing trends have shifted from legacy PFAS to shorter-chain alternatives that are suggested to be safer, such as perfluorohexanoic acid (PFHxA). However, the current amount of data to support safety assessments of these alternatives is not yet sufficient. The present study investigated the effects of a 42-day dietary exposure to 1, 10, or 100 ng/g PFHxA in juvenile zebrafish. The zebrafish model was leveraged to interrogate morphometrics, fecundity, and numerous behavior endpoints across multiple generations. Dietary PFHxA exposure did not result in measurable body burden and did not affect growth, fecundity, adult social perception behavior, or associative learning. PFHxA exposure did induce abnormal adult anxiety behaviors in the F0 generation that persisted transgenerationally in the F1 and F2. Abnormal larval and juvenile behavior was observed in the F1 generation, but not in the F2. PFHxA juvenile dietary exposure induced subtle and multigenerational behavior effects that warrant further investigation of this and other alternative short-chain PFAS.

## 1. Introduction

Per- and polyfluoroalkyl substances (PFAS) are ubiquitous anthropogenic contaminants of increasing concern due to associated adverse health effects. PFAS are a broad chemical class in which strong carbon-fluorine bonds impart highly desirable properties for a variety of applications (e.g., stability, low chemical reactivity, heat resistance and conductivity, high hydrophobicity and oleophobicity, and ability to lower surface tension) [1]. PFAS are commonly used as processing aids (e.g., in the manufacturing of fluoropolymers and polyethylene films) and in a variety of industrial and consumer products, such as cookware, food packaging, personal care products, clothing and textiles, paints and inks, electronics, cleaning products and waxes, medical utensils, and fire-fighting foams [1,2]. Extensive production, use, and disposal have led to ubiquitous detection of PFAS in environmental media, along with high frequency of detection among human populations worldwide [3,4,5,6].

While inhalation and dermal absorption of PFAS are increasingly recognized as significant routes of exposure, diet and drinking water are the predominate routes [7,8,9,10]. Studies of adult populations in industrial countries within the northern hemisphere indicate that dietary exposure contributed >90% of the total intake of perfluorooctane sulfonate (PFOS) and perfluorooctanoic acid (PFOA), the most extensively studied PFAS [11]. Furthermore, approximately 40–85% intake of a range of perfluoroalkyl carboxylic acids resulted from dietary exposure [12]. PFAS can migrate into food products from packaging, be taken up by agricultural crops following treatment with contaminated water or biosolids, and bioaccumulate in animal products [7]. Widespread exposure is particularly concerning as a number of PFAS have been shown to detrimentally impact thyroid function and the immune system, lead to liver and kidney disease, and cause adverse reproductive and developmental outcomes in a variety of model systems [13].

Toxicity data and public awareness have invoked regulatory action and voluntary phase outs of legacy compounds in several countries over the past two decades. Phase outs of PFOS and PFOA have led to declining concentrations in human populations, as primarily measured in serum [6,14]. Such initiatives have also led to a shift in manufacturing trends towards alternative, often shorter-chain PFAS [15]. Typically defined as those with 4–6 fully fluorinated carbons [16,17], short-chain PFAS are thought to be safer alternatives. Unfortunately, the amount of safety data on shorter chain replacements are currently inadequate. Exposure to short-chain compounds will surely increase with increasing production, emission, and continued degradation of longer PFAS (e.g., fluorotelomer alcohols and fluorotelomer phosphate diesters) into terminal degradation products [15,18]. The increased hydrophilicity of short-chain compounds compared to their long-chain counterparts reduces bioaccumulative potential in animals and humans [15,17]. Nevertheless, short-chain PFAS are still highly persistent in the environment and bioaccumulate in agricultural crops, fruits, and vegetables. In some cases, affinity for accumulation is greater than longer-chain homologues [19,20,21]. Short-chain compounds are also increasingly detected in breast milk and at levels comparable to PFOS and PFOA [22,23]. Given the potential for human exposure through diet, limited safety data, and current knowledge of their toxicology, investigation of the health effects of short-chain PFAS following dietary exposure is essential.

Perfluorohexanoic acid (PFHxA) is a short-chain PFAS frequently detected in the environment, specifically in drinking water and foodstuffs [24]. In crops sampled in 2014 from fields located 0.3 km from a mega-fluorochemical industrial park in Shandong Province, China, PFHxA concentrations ranged from 1.28 to 197 ng/g [20]. In the most consumed foods of the general population in Taipei City, Taiwan from 2010–2011, one study indicated 100% detection of PFHxA in nearly all food items, with mean concentrations ranging from 0.90 to 1.58 ng/g [25]. Others have also reported frequent PFHxA detection in food and at comparable concentrations [26]. Significant risk of exposure, predominately dietary, creates concern that continuous exposure to background concentrations of PFHxA may cause adverse health effects [15].

PFHxA has low acute toxicity in animal bioassays; however, studies have shown a variety of sublethal effects following sub-chronic and chronic exposure in rodents [27] and following developmental exposures in zebrafish [28,29,30]. Due to the limited number of epidemiological studies that have addressed health effects of PFHxA exposure, and the cross-sectional nature of these studies, it is necessary to rely on available animal bioassays (Luz 2019). Rodent studies, predominately oral gavage exposures of adults, have reported no significant reproductive, developmental, or locomotor effects, but mixed effects on body weight gain, serum chemistry, and target organ toxicity [27]. Though a number of rodent toxicity studies indicated that PFHxA was significantly less toxic than PFOS and PFOA, additional assessment is still necessary to confidently declare its safety. For instance, developmental toxicity in the form of abnormal locomotor behavior has been observed following PFHxA exposure in several developmental zebrafish studies [28,29]. Sublethal effects of PFHxA exposure have not been adequately investigated. In particular, the sensitivity of juveniles to dietary exposure, behavior endpoints, and the potential for transgenerational effects require further investigation.

Traditional rodent studies are effort-, time- and cost-intensive, whereas alternative models such as zebrafish enable more rapid testing of chemical impacts on numerous sensitive, sublethal endpoints, still in a complex biological system. Relevant to both environmental and human health, zebrafish are a popular model extensively used for toxicological studies [31]. Zebrafish are amenable to husbandry in laboratories, boast high fecundity, share significant genetic homology with humans [32], and enable investigation of transgenerational effects [33]. The present study leveraged the zebrafish model to investigate health effects of dietary exposure to PFHxA in juvenile zebrafish during a critical period of gonadal development. Effects immediately following exposure, later in adulthood, and in subsequent generations were interrogated. Evaluating relevant diet concentrations previously reported in food and utilizing the sensitivity of zebrafish behavior endpoints across life stages and generations, this study contributes to our knowledge of PFHxA toxicity.

## 2. Materials and Methods

### 2.1. Zebrafish Husbandry

Wildtype Tropical 5D zebrafish (*Danio rerio*) were bred and raised at Oregon State University in the Sinnhuber Aquatic Research Laboratory (SARL) [34]. All protocols followed guidelines approved by Oregon State University’s Institutional Animal Care and Use Committee (protocol 2020-0136). Under a 14:10 h light:dark cycle, fish were maintained at 28 °C and housed at densities of 500 fish per 50 gallon tank with recirculating filtered water supplemented with Instant Ocean salts. Spawning initiated at 8 AM, when the lights turned on, and embryos were collected over the span of an hour using spawning funnels that were placed in the tanks the night prior. Following collection, embryos were kept in plastic petri dishes at 28 °C in embryo medium (EM) consisting of 15 mM NaCl, 0.5 mM KCl, 1 mM MgSO_4_, 0.15 mM KH_2_PO_4_, 0.05 mM Na_2_HPO_4_, and 0.7 mM NaHCO_3_ [35]. Embryos were surface sanitized with sodium hypochlorite at 6 h post fertilization (hpf) [34,36] and maintained in EM with methylene blue in petri dishes until 5 days post fertilization (dpf). At this time, larvae were transferred to 2.8 L tanks at densities of 100 fish per tank, put on a constant flow-through system, and fed uncontaminated GEMMA Micro 75 pellets from 5 to 16 dpf.

### 2.2. Chemicals and Preparation of Stock Solution

PFHxA (CAS: 307-24-4) was obtained from Matrix Scientific (Columbia, SC, USA; Lot: L08T). Approximately 0.2 g was dissolved into 25 mL methanol (Fisher Scientific, Waltham, MA, USA; LC/MS grade; CAS: 67-56-1) in a 30 mL polypropylene bottle and shaken on an orbital shaker overnight to achieve a target 30 mM concentration, as previously reported by Rericha et al., 2021. An aliquot from this stock solution was diluted and used for analytical measurement of stock concentration by high-performance liquid chromatography and triple quadrupole mass spectrometry (LC-MS/MS) [29] and for zebrafish diet contamination. The measured concentration of the stock solution was utilized to calculate necessary dilutions throughout the study (see Sections S1–S3 for additional analytical information).

### 2.3. Top Coating of Zebrafish Diet with PFHxA

Diets targeting nominal concentrations of 0, 1, 10, and 100 ng/g PFHxA were prepared via top coating for two diet granule sizes obtained from Skretting (Stabanger, Norway): GEMMA Micro 75 (50–100 µm pellet size; Lot: 7260181) and GEMMA Micro 150 (10–200 µm pellet size; Lot: 5188098). The two granule sizes were necessary to accommodate the needs of growing zebrafish; Both diets consisted of 59% protein, 14% oil, and 14% ash. To make each treatment, 50 g of diet was thinly spread in a circular cake tin lined with aluminum foil. PFHxA stock solution was diluted into 40 mL of methanol inside of 50 mL high density polyethylene (HDPE) spray bottles (VWR; Cat: 10216-890) to achieve the appropriate mass for each target nominal concentration. The solution was sprayed onto the diet until the surface was evenly damp, and the diet was mixed thoroughly with a stainless-steel spatula, allowed to dry, and respread across the tin. This process was repeated until the contents of the bottle were depleted, at which point the spray bottle was rinsed thrice with 3 mL of methanol and rinsate was sprayed onto the diet. Following complete application, the diet was allowed to dry for 2 h and passed through a 1 mm sieve. The PFHxA-contaminated diet was thoroughly mixed to ensure homogeneity, distributed into 30 mL polypropylene tubes (VWR; Cat: 89012-778) and stored at 4 °C. The control diet was prepared using the same method, but with only methanol. Triplicate samples of each diet were collected and stored at −20 °C until analytical verification of PFHxA concentration using LC-MS/MS (see Sections S1–S4). Average measured concentrations in replicate (*n* = 2 or 3) diet samples varied from 51–310% of nominal (Table 1). However, the approximate desired breadth of concentrations was achieved, and there was sufficient consistency in measured PFHxA concentrations between the diet granule sizes for each nominal concentration. For clarity throughout the remainder of this study, the dietary exposures are reported as the nominal concentration.

### 2.4. Dietary Exposure

**Figure 1:**
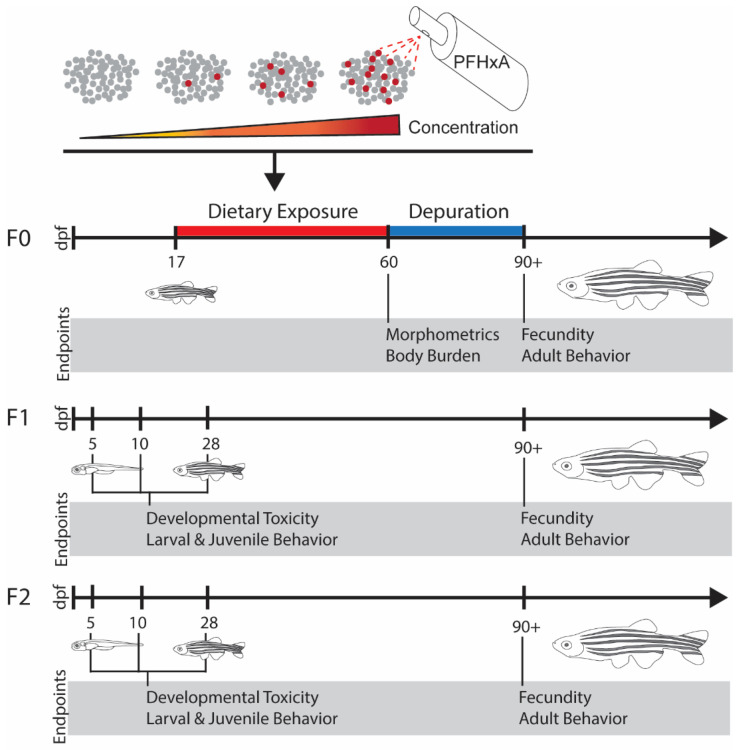
Experimental Overview. F0 zebrafish were dietarily exposed from 17 to 59 days post fertilization (dpf) to diets contaminated with (left to right) 0 (control), 1, 10, or 100 ng/g PFHxA. Immediately following dietary exposure, measurements of morphometrics and body burden were performed. Following a subsequent depuration period, fecundity and adult behavior were assessed after 90 dpf. One spawning event was used to generate the F1 generation, which underwent assessments of developmental toxicity, larval and juvenile behavior, fecundity, and adult behavior. The same experimental protocols were used to interrogate transgenerational effects in the F2 generation.

At 17 dpf, the juvenile zebrafish were transferred to 8 tanks of 9 L capacity at densities of 120 fish per tank and put onto an isolated intermittent flow system to facilitate the dietary exposure. Fish were fed the control or PFHxA-contaminated diets from 17 to 59 dpf, a total of 42 days encompassing critical windows of gonad development (Figure 1) [37]. From 17 to 30 dpf, exposure groups in two tanks each (*n* = 240 fish total) were fed the control or contaminated GEMMA Micro 75 diets. Each tank received an average of 63 mg per tank (1/32 tsp scoop) three times per day to facilitate normal feeding behavior. At 31 dpf, each tank was split into two for a total of four groups of four 9L tanks, each at a density of 50 fish per tank. From 31 to 59 dpf, the exposure groups were fed control or contaminated GEMMA Micro 150 diets. The amount fed to the tanks was adjusted as the fish grew. An average of 69 mg (1/32 tsp scoop) was fed three times per day from 30 to 45 dpf, and an average of 103 mg (1/32 tsp plus 1/64 tsp scoops) from 45 to 59 dpf. At 50 dpf, fish were only fed twice per day. Owing to the small size of the fish and feeding behavior during the juvenile life stage, it was not possible to quantify the amount of food consumed. However, analytical validation of the contaminated diets provided confidence that consumed food was contaminated at target concentrations. Additionally, water inputs were scheduled within 15–60 min after each feeding to remove uneaten food and circulate water. Tanks received 8 water inputs per day, consisting of 3.8–4.8 L of system water per input to reach a total of 3–4 tank exchanges per day during the dietary exposure period. Detritus was removed from tanks weekly via siphoning, and water quality (i.e., temperature, pH, ammonia, nitrate, and nitrite levels) was monitored daily (Table S2).

At 60 dpf, a subset of fish was sacrificed for weight and length measurements, also sampling for analytical measurement of body burden and organs. Prior to the sampling, tanks were fasted overnight to minimize the risk of PFHxA contamination of samples from food remaining in the gut. Following the sampling, remaining fish were maintained in the isolated intermittent flow system and fed uncontaminated GEMMA 300 (200–500 µm pellet size; Lot: 7268495) for a depuration period from 60 to 90 dpf. Water inputs increased during this time to 5–6 L each to maintain proper water quality conditions as the fish grew. At 90 dpf, following the completion of the depuration period, fish were transferred back to the constant flow-through system, where they were housed for the remainder of the study at densities of 16 fish per 2.8 L tank [34].

### 2.5. Morphometric Measurements and Body Burden

Immediately following completion of the dietary exposure at 60 dpf, morphometric measurements of length, body weight, and organ weights were performed to assess exposure effects on juvenile growth and development. Prior to measurements and dissections, fish were euthanized by hypothermic shock. Euthanized fish were dipped in 90% ethanol solution and standard length (snout to caudal peduncle) was measured (*n* = 36) [38]. Fish were gently dried with a paper towel and then weighed (*n* = 36). Fulton’s condition factor *K* was calculated as a measure of weight-length relationship following the below equation where *W* is wet weight in g and *L* is length in cm [39]:

(1) $$K=100\frac{W}{L^{3}}$$

Effects on length, weight, and *K* were determined using a one-way ANOVA (*p* < 0.05), or a Kruskal-Wallis test (*p* < 0.05) if ANOVA assumptions were not met (Shapiro-Wilk and Levene tests). Throughout this study, all visualizations and statistical analyses were performed using R version 4.1.2.

Following length and weight measurements, whole fish (*n* = 3) were collected into 1.5 mL safe-lock tubes (Eppendorf; Cat: 022363204; Hamburg, Germany), snap frozen in liquid nitrogen, and stored at −80 °C for analytical measurement of body burden using LC-MS/MS (Sections S1–S4), and the rest were dissected to sample the brain and liver. Dissections were conducted in two blocks to counteract time effects between exposure groups, and dissection equipment was cleaned with ethanol between groups. Per exposure group, 4 replicates of 8 pooled samples of each organ were collected in safe-lock tubes, weighed by difference, and then flash frozen before storage at −80 °C. Diet effect on brain and liver weight were evaluated using a one-way ANOVA (*p* < 0.05). Intestines were collected individually for analysis in future studies.

### 2.6. Fecundity Assessments

To evaluate dietary exposure effects on fecundity, spawning groups (*n* = 4) consisting of 3 males and 2 females were housed in 1.8 L tanks. Each spawning group was spawned approximately every 10 days for 5 consecutive events between 90 and 140 dpf. The night before each spawning event, fish were placed into 1.0 L crossing tanks (Aquaneering; Cat: ZHCT100; San Diego, CA, USA) with the males and females separated by gates. In the morning, at 8:00 am when the lights turned on, gates were removed, water flow was halted, and fish were allowed to spawn for 1 h. Embryos were collected and maintained in EM in plastic petri dishes. Between 4–6 hpf, embryos were assessed, sorted, and counted as fertilized, unfertilized, or necrotic. Due to natural variability in spawning, all fecundity data was analyzed without consideration of spawning event date (*n* = 20 per exposure group). Differences in the total number of embryos and the number of fertilized embryos between exposure groups were determined using a Kruskal-Wallis test followed by Dunn’s post-hoc (*p* < 0.05).

After select spawning events, embryos underwent developmental toxicity assessments or were surface sanitized and raised to produce F1 and F2 generation populations for evaluation of multigenerational effects. Approximately 300 fish per exposure group and generation were housed on the flow through system at densities of 100 fish per 2.8 L tank, until 30 dpf when densities were adjusted to 16 fish per tank. Fecundity assessments using the same protocol used for the F0 generation were also conducted for the F1 and F2, with the sole exception of conducting 6 consecutive spawning events for the F1.

### 2.7. Adult Behavior Assessments of F0, F1, and F2 Generations

We utilized several adult behavior assays interrogating social interaction (shoaling), normal locomotion (free swim), predator, schooling, and startle response, and associative learning to investigate whether juvenile dietary exposure altered behavior later in life at 90+ dpf. All adult behavior assays were conducted within 3 weeks with the same cohort of fish. These assays were performed for the F0 exposure groups that were fed PFHxA-contaminated diet, and also for the F1 and the F2; assessment of the F2 generation enabled investigation of transgenerational effects.

#### 2.7.1. Shoaling

To assess social interactions, we evaluated 16 groups of 4 zebrafish (2 males and 2 females) for each exposure group (*n* = 64 total). Each group was placed into a 1.7 L tank (26 × 5 × 12 cm) and recorded for 30 min of uninterrupted swim. Tracking using PhenoRack (ViewPoint Behavior Technology, Lyons, France; version 5.27.7.70) software enabled measurement of three endpoints every 30 s: inter-individual distance (IID), the average distance between all the fish while shoaling; nearest-neighbor distance (NND), the average distance between the two nearest fish in the shoal; and average speed. The initial 25 min of the assay was an acclimation period, and statistical analysis was restricted to the last 5 min. For each fish, the endpoints from each time bin were aggregated by mean. Statistical differences between exposure groups were identified using a one-way ANOVA followed by a Tukey post-hoc test (*p* < 0.05) for each endpoint. Following the shoaling assay, fish were returned to normal housing until further testing.

#### 2.7.2. Free Swim

Locomotion was assessed using an individual free swim assay that utilized the same tank and duration as the shoaling assay. Free swim evaluated one fish per tank (*n* = 63–68, 32–35 males and 31–34 females), generating data on speed and distance traveled for every 30 s time bin. The beginning of the assay was an acclimation period and only the last 5 min were analyzed, aggregating the mean total distance from time bins for each fish. Prior to statistical analysis, any fish with a mean total distance of 0 cm were removed from the data to ensure that instances of poor tracking were not considered. To determine differences between groups, a two-way ANOVA considering exposure group and sex followed by a Tukey post-hoc test were performed (*p* < 0.05).

#### 2.7.3. Predator, Schooling, and Startle Response

The predator, schooling, and startle response assays were conducted using the zebrafish visual imaging system (zVIS) [33]. Eight tanks (10 × 10 × 13 cm) were arranged in an array so that one side of each tank views a video projection [40]. Fish were recorded by a camera mounted above the array and EthoVision 11.5 XT tracking software (Noldus) was used to track fish and total movement in 1 s time bins. Individual fish (*n* = 56–68, 28–34 males and 27–34 females) were placed into the tanks in 750 mL of water and recorded during the 37 min assay that was divided into four time periods: acclimation (0–20 min), predator response (20–25 min), second acclimation (25–30 min), and schooling response (30–35 min), followed by startle response (2 min). During acclimation periods, fish were allowed to swim in the tanks without any external stimulus. For the entirety of the predator response period, a video of a predator fish was shown [33]. During the schooling response period, a video of schooling zebrafish was shown. After, the startle response period consisted of 10 consecutive taps every 20 s. The tap stimulus was generated underneath the tanks by an electric solenoid, each fired simultaneously under all the tanks [33].

For analysis of both the predator and schooling responses, 3 zones of equal area were defined within each tank: close (next to the video projection), middle, and far (farthest from video projection). Percent time spent in the near zone was assessed, considering 1 min before and 1 min of the video. Differences in the percentage of time spent in the near zone between exposure groups were determined using three-way ANOVAs considering exposure group, sex, and video status (i.e., before or after video projection) and Tukey post hoc tests (*p* < 0.05). For startle response, habituation across the first 5 taps was interrogated using a three-way repeated measure ANOVA, with resampling for each tap. Differences in mean distance traveled between exposure groups was analyzed using a three-way ANOVA and Tukey post hoc tests (*p* < 0.05).

#### 2.7.4. Associative Learning

Effects of juvenile dietary exposure on adult associative learning were evaluated using a shuttle box assay. Shuttle box design and assay setup followed protocols established by Truong et al. [41]. Briefly, shuttle boxes (200 × 100 × 90 mm) contained an internal divider at the midpoint of the length with a 10 mm gap along the floor, to enable fish to shuttle between the two sides, and a pair of infrared light beams to monitor which side of the shuttle box the fish was in. Boxes were filled with 250 mL of water and individual fish (*n* = 62–66, 30–33 males and 30–33 females) were allowed to swim freely throughout the box. A conditioning stimulus (blue light) meant to prompt the fish to swim to the other side of the shuttle box was achieved by LED light, and an unconditioned stimulus (mild electric shock of 3.0 volts) was created between stainless steel plates at either end of the box when the fish remained on the incorrect side. A series of consecutive trials entailed a 600 s acclimation period followed by 32 consecutive trials lasting 24 s each, with an intertrial period of 60 s. During each trial, upon presentation of the conditioning stimulus, fish were allowed an 8 s decision period. If fish did not swim to the other side of the shuttle box, the unconditioned stimulus was administered until the desired outcome was achieved or until 16 s passed. Fish were considered “learners” if 8 consecutive trials were achieved without triggering the unconditioned stimulus, and the critical trial number (i.e., the first of the 8 consecutive trials) was reported. For fish that did not exhibit associative learning and failed to achieve the desired outcome (i.e., were shocked for 16 s) for 8 consecutive trials, the assay was terminated. Differences in the number of learners between groups were determined using Chi-squared tests considering exposure group and sex (*p* < 0.05). Exposure and sex effects on the critical trial number of the learners (i.e., the first trial of at least 8 successful consecutive trials) was also investigated using two-way ANOVAs and Tukey post hoc test (*p* < 0.05).

### 2.8. Developmental Toxicity Assessments of F1 and F2

To assess developmental toxicity in subsequent generations, two cohorts of F1 embryos or F2 embryos obtained during F0 or F1 fecundity assessments were evaluated for morphology and behavior endpoints. For each offspring cohort, fertilized embryos (*n* = 48) were randomly sampled from all 4 spawning sets within each exposure group and generation and placed into 96-well plates (Falcon; Cat: 353227) containing 100 µL EM per well. Plates were covered with parafilm to minimize evaporation and maintained in the dark at 28 °C until evaluation.

At 24 hpf, embryos were evaluated for mortality, delayed development, and spontaneous movement. At 120 hpf, larval behavior was evaluated with a larval photomotor response (LPR) assay using ViewPoint Zebraboxes and video tracking software [42,43]. The software tracked each embryo with a data bin time of 6 s. The assay consisted of an initial 6 min acclimation period followed by 4 cycles of 3 min of light (2300 Lux) and 3 min in the dark (IR), only the last cycle of which was analyzed. Statistical analysis followed the workflow described by Zhang et al., 2017. Briefly, using a custom R script, total distance moved was plotted, dead or malformed fish were excluded from the analysis, differential entropy was modeled, and statistical significance was evaluated based on the area under the curve ratios for treated versus control groups using a Kolmogrov-Smirnov (KS) test [44]. A visual mortality assessment and evaluation of 9 morphological endpoints followed the 120 hpf behavioral assay (Table S3A for all endpoint descriptions and Table S3B for additional information).

### 2.9. Juvenile Behavior Assessments of F1 and F2 Generations

Subsets of fish from the F1 and F2 generations produced following select spawning events were sampled for juvenile behavior assessment using three assays. All juvenile assays were recorded using Zebraboxes and analyzed using PhenoRack software.

#### 2.9.1. Light/Dark Preference

Early in juvenile development, at 10 dpf, we used a light/dark preference assay to identify abnormal behavior. The assay apparatus consisted of a plate with 8 rectangular wells (31 × 42 × 18 mm), with half of each well consisting of a dark zone created by securing visibly opaque but IR transparent film underneath the plate and the other half of each well consisting of a lighted zone without film, as adapted from Shen et al. [45]. Individual fish (*n* = 31–40) were placed into 5 mL of water in each well. The assay lasted 12 min, consisting of first an acclimation period in the dark (0–6 min; IR) and then a light period (6–12 min; 550 Lux) during which visible light from underneath the plate stage created obvious light and dark zones. Total distance was calculated for every 30 s time bin, and after aggregating by mean, fish that had an average total distance of 0 for the entire dark acclimation period were removed from the analysis. Within the light period, the first 3 min were considered additional acclimation, and the last 3 min were analyzed. Total distance traveled and percent time spent in each zone were evaluated by aggregating means for each fish, and differences between control and treated groups were determined using a two-way ANOVA considering exposure group and zone, with a Tukey post-hoc test (*p* < 0.05).

#### 2.9.2. Mirror Response

To investigate early social behavior at 28 dpf, we conducted a mirror response assay. The assay utilized a 24-well plate in which each well (15 × 15 mm) contained a mirror on one side [45]. The third of the well closest to the mirror was defined as the mirror zone. One fish was placed into 1 mL of water in each well (*n* = 48). The duration of the assay was 10 min, broken into a dark acclimation period (0–5 min; IR) followed by a light period (5–10 min; 550 Lux) during which the fish could see their reflection in the mirror. Percent time and distance traveled in the mirror zone were calculated for every 30 s time bin, fish with an average total distance of 0 for the dark period were removed, and then the last two min of the dark period and the last two min of the light period were analyzed. Endpoints were aggregated by mean for each fish. Significant differences between exposure groups were determined using a two-way ANOVA considering exposure group and period, followed by a Tukey post-hoc test (*p* < 0.05).

#### 2.9.3. Juvenile Shoaling

The juvenile shoaling assay was conducted at 28 dpf as an additional investigation of social behavior effects [45]. The assay assessed groups of 4 fish in one arena (125 × 81 × 40 mm). 12 groups of 4 fish per exposure group were evaluated by placing fish into the arena with 5 mL of water and allowing uninterrupted swim for 7 min. Just as for the adult behavior shoaling assay, IID, NND, and speed measurements were calculated in 1 min time bins. Analysis was conducted by aggregating each endpoint by mean for each group of fish. Differences in average IID, NND, and speed between exposure groups were determined using a one-way ANOVA followed by a Tukey post-hoc test (*p* < 0.05).

## 3. Results

### 3.1. PFHxA Was Not Detected in Whole Body Samples following Dietary Exposure and Did Not Affect Growth or Morphometrics

**Figure 2:**
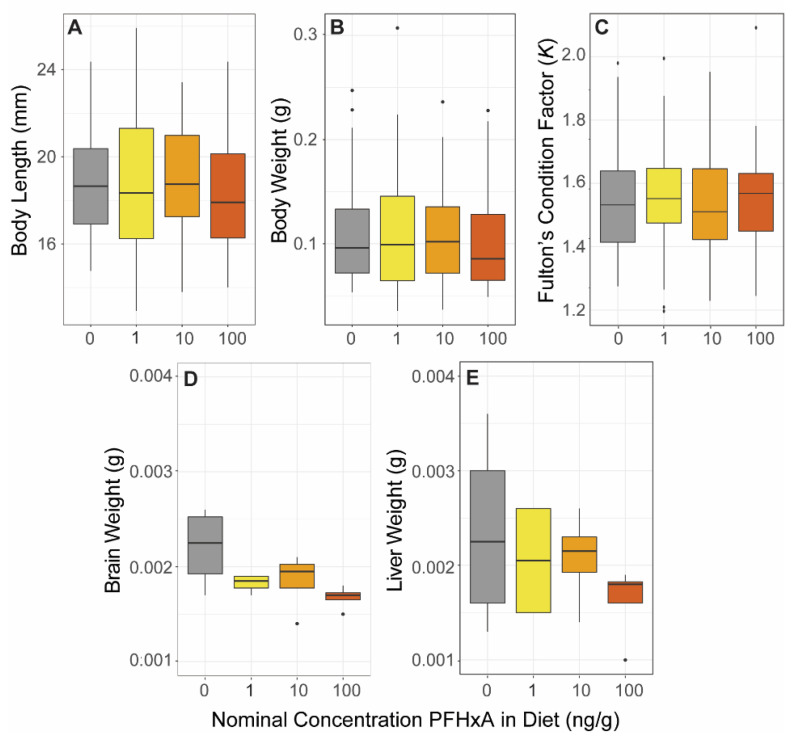
Morphometrics. At 60 dpf, following dietary exposure, zebrafish body length (**A**), weight (**B**), and Fulton’s Condition Factor *K* (**C**) were measured (*n* = 36), as well as average weight (*n* = 4) of 8 pooled brains (**D**) or 8 pooled livers (**E**). No significant differences between exposure groups were observed.

PFHxA concentrations were below the limit of detection for 60 dpf whole body samples collected immediately following the 42 days of dietary exposure (*n* = 3). For this reason, and the small mass of the pooled organ samples, tissue burdens of the liver and brain were not analytically measured. Evaluation of morphometrics at the 60 dpf timepoint revealed normal growth and development. No statistical differences between control and exposure groups were observed for length or body weight (Figure 2). Length-weight relationship, as assessed using Fulton’s Condition Factor (*K*), showed no exposure group effect. Pooled average brain and liver weights were also not affected. Overall, dietary exposure to PFHxA did not alter growth and development as measured in the present study.

### 3.2. Dietary Exposure to PFHxA Did Not Impact Fecundity for F0 or F1, though an Exposure Group Effect Was Noted for the F2

**Figure 3:**
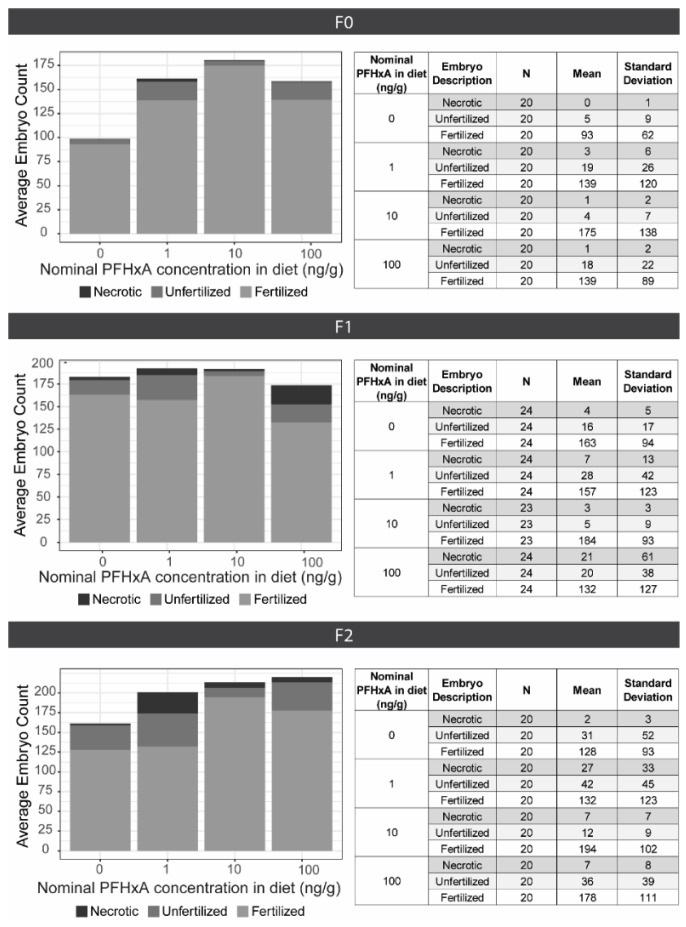
Fecundity Assessments. 4 groups of 3 male and 2 female zebrafish were spawned for 5–6 consecutive spawning events per generation (top: F0, middle: F1, bottom: F2). The average embryo counts are plotted on the left, including necrotic (black), unfertilized (dark grey), and fertilized (light grey) embryos for each exposure group (0, 1, 10, or 100 ng/g PFHxA added to the F0 diet). The tables on the right indicate the evaluated number of spawns (N), mean counts, and standard deviation.

Assessed over a series of 5–6 spawning events per generation, dietary exposure did not impact the fecundity of either the F0 or subsequent generations. Within each generation, the total number of embryos produced did not significantly differ across groups (Figure 3). The number of fertilized embryos within the F0 or F1 generations was not affected by dietary exposure, but was in the F2 generation (*p* = 0.049); however, no differences between control and exposed groups were identified by the Dunn’s post-hoc test. Naturally occurring variability between spawning groups and spawning events among all the exposure groups led to high standard deviation and significant overlap in embryo counts.

### 3.3. Aberrant Adult Behavior Observed in the F0, F1, and F2 Generations following F0 Juvenile Dietary Exposure to PFHxA

#### 3.3.1. F0 Generation

**Figure 4:**
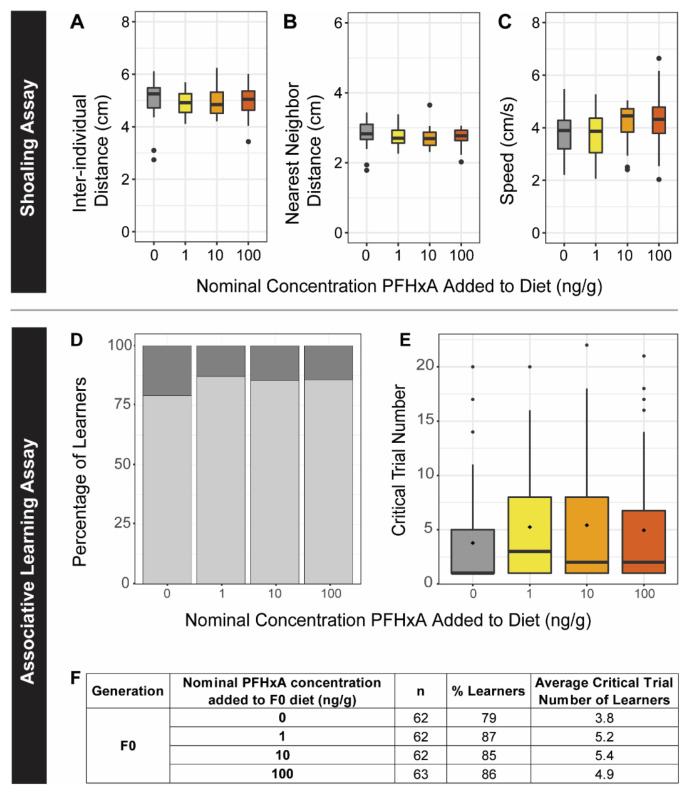
Social Perception and Associative Learning Behavior for F0. F0 zebrafish were challenged with adult behavior shoaling (**A**–**C**) and associative learning assays (**D**–**F**). Exposure groups are indicated on the x-axes and by color (left to right, grey: 0 ng/g, yellow: 1 ng/g, orange: 10 ng/g, and red: 100 ng/g PFHxA added to the diet). Dietary exposure did not alter social perception (*n* = 64), as measured by inter-individual distance (**A**), nearest neighbor distance (**B**), or speed (**C**). Learning was also not impaired by PFHxA exposure, as measured by the percentage of learners (**D**) and critical trial number (**E**). Sample size, percent learners, and mean critical trial are presented in the table (**F**).

The dietarily exposed F0 generation was evaluated for learning impairment, social, and anxiety behaviors. To measure social perception (shoaling assay), adult shoaling behavior was evaluated using 3 parameters: inter-individual distance (IID), nearest-neighbor distance (NND), or speed (Figure 4). None of the exposure groups exhibited changes in social perception. To determine if dietary exposure to PFHxA altered learning, F0 fish were tested in an associative learning assay using a custom-built shuttlebox. Over 30 consecutive trials, the fish were conditioned to associate light with a lack of adverse event (mild shock), therefore resulting in fish moving towards the light. After 8 consecutive trials of not being shocked, a fish was deemed a learner, and the first of the 8 successful trials was noted as the critical trial number. The number of learners within the exposure groups was not significantly different and the critical trial number was not impacted relative to the controls (Figure 4), though there were differences in the number of learners based on sex (*p* = 0.035) (Table S4). Overall, PFHxA dietary exposure did not affect measured learning or social behavior in the F0 generation.

**Figure 5:**
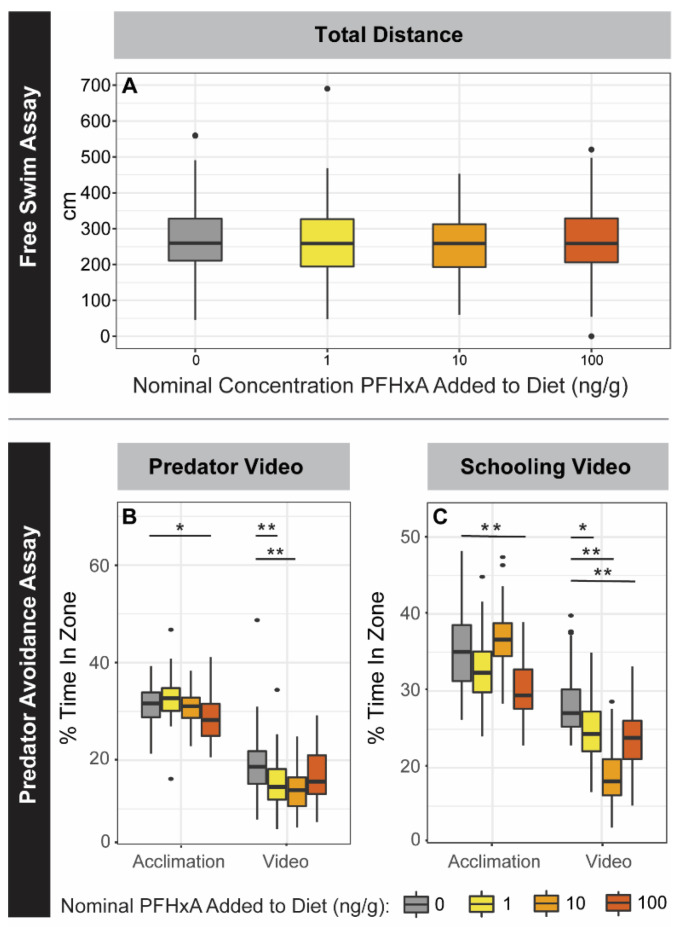
Anxiety Behaviors for F0. F0 zebrafish were challenged with adult behavior free swim (**A**), predator response (**B**), and schooling response assays (**C**). Exposure groups are indicated on the x-axes and by color (left to right, grey: 0 ng/g, yellow: 1 ng/g, orange: 10 ng/g, and red: 100 ng/g PFHxA added to the diet). PFHxA juvenile dietary exposure did not affect the total distance traveled in the free swim assay (*n* = 66–68). The predator and schooling response plots show the percent time (cumulative duration) spent in the near zone (i.e., closest to the video display). For both predator and schooling response (*n* = 65–68), time spent was significantly decreased for all groups after video display (Video) compared to before (Acclimation). Significant differences between exposure groups within the Acclimation and Video periods are indicated on the plots (* *p* < 0.05; ** *p* < 0.005).

To assess anxiety-like behavior, zebrafish unstressed swim pattern in isolation (free swim), and predator, schooling, and startle response were measured. For the free swim assay, the fish were placed in an unstressed environment for 30 min, and no significant change in swimming distance was measured relative to the control (Figure 5), including when separated by sex (Figure S1). During the predator and schooling response assays, videos of a predator fish or schooling zebrafish were shown and time spent in the zone nearest the video projection was measured. Percent time spent in the near zone decreased for all exposure groups after the predator video, but the 1 and 10 ng/g F0 groups spent less time compared to controls (*p* < 0.05), exhibiting greater predator avoidance behavior (Figure 5). Separating by sex, 10 and 100 ng/g group females displayed increased predator avoidance behavior relative to sex-matched controls, whereas the 100 ng/g males decreased in predator avoidance behavior (Figure S2; Data S1). Counter to expectations, the schooling response video invoked a similar avoidance behavior to the predator video; all exposure groups spent less time in the near zone following the schooling video (Figure 5). The 1, 10, and 100 ng/g F0 groups showed greater avoidance behavior than controls, though the 100 ng/g group spent significantly less time in the near zone relative to controls before the schooling video as well (Data S4). Finally, for the startle response assay, the 100 ng/g PFHxA F0 group exhibited a hyperactive startle response compared to the control group (*p* = 0.035) following a series of solenoid taps (Figure S3; Data S7). Subtle, statistically significant abnormalities in anxiety behaviors during the predator, schooling, and startle response assays were evidence that dietary exposure across the tested concentration range affected F0 development.

#### 3.3.2. F1 Generation

The F1 generation was subsequently evaluated for learning impairment, social, and anxiety behaviors to determine whether dietary exposure to the F0 caused multigenerational effects. As for the previous generation, F1 groups exhibited predominately normal social perception in the shoaling assay, with no changes in IID or NND relative to controls. However, speed was significantly higher by 0.9 cm/s in the 1 ng/g lineage compared to controls (*p* = 0.024; Figure S4). Similarly, there was no observed impairment in associative learning in any exposure group, though the average critical trial number was affected by sex (*p* = 0.040; Table S4).

Like the F0 generation, aberrant anxiety behaviors were observed for F1 only during the predator, schooling, and startle response assays, but not in the free swim assay (Figures S1 and S5). For the predator response assay, the F1 generation was characterized by higher magnitude differences between the exposure group and control lineages before display of the predator video (Figure S6). For example, the 1 ng/g group spent 10% more time in the near zone relative to controls largely driven by male individuals (Figure S2; Data S2), and this preference was maintained following the video display. Despite initial differences in behavior, for all exposure groups, the percent time spent in the near zone decreased following predator video display, as expected for predator avoidance behavior. Additional sex-driven behavior differences were observed, with exposure lineage males displaying greater near zone preference than controls (Figure S2). During the schooling assay, the F1 exposure lineages preferred the near zone to a greater extent than controls regardless of video display, which was dissimilar to F0 behavior (Figure S6; Data S5). Despite this preference, the 1 and 10 ng/g groups still exhibited avoidance behavior following the video display, maintaining response trends observed in the previous generation. The F0 100 ng/g exposure group exhibited hyperactivity in the startle response assay, while the 1 and 10 ng/g lineages displayed hyperactive startle response in the F1 generation (Figure S3; Data S8). Though the presence of abnormal anxiety behavior in the predator, schooling, and startle response assay was consistent between the F0 and F1 generations, the responses, and impacted groups varied.

#### 3.3.3. F2 Generation

To investigate the transgenerational effects of F0 dietary exposure to PFHxA, the F2 generation was challenged with the same adult behavior assays to interrogate learning, social, and anxiety behaviors. As was true for both previous generations, the F2 exposure group lineages exhibited normal social perception (shoaling assay) and associative learning (Figure S4; Table S4).

F2 anxiety-related behavior trends were highly similar to those observed in the F1 generation. No change in swimming distance was noted during the free swim assay (Figure S5). In the predator response assay, decreased time spent in the near zone for all F2 groups following the video display was evidence of the expected avoidance behavior (Figure S6). The 1 ng/g group exhibited a greater preference for the near zone compared to controls both prior to and during the predator video display. Similar to the F1 generation, more instances of abnormal behavior were observed for exposure lineage males than females in the F2, particularly from the 1 and 10 ng/g groups (Figure S2; Data S3). Fewer overall effects were observed during the schooling response assay in the F2 generation (Figure S6). Only the 1 ng/g group spent increased time in the near zone compared to controls both before and during the schooling video, and only the 1 and 10 ng/g groups exhibited avoidance behavior after the schooling video (Figure S6; Data S6). Unlike the F0 and F1, sex was a significant factor for F2 schooling response. During the startle response assay, the 1 and 10 ng/g F2 groups exhibited hypoactivity, in contrast to the hyperactivity observed in the F0 100 ng/g group and the F1 1 and 10 ng/g groups (Figure S3; Data S9). Altered anxiety behavior measured in the predator, schooling, and startle response assays are evidence of transgenerational effects resulting from F0 dietary exposure.

### 3.4. F0 Exposure Induced Aberrant Behavior during Development of Larval F1 but Not F2

**Figure 6:**
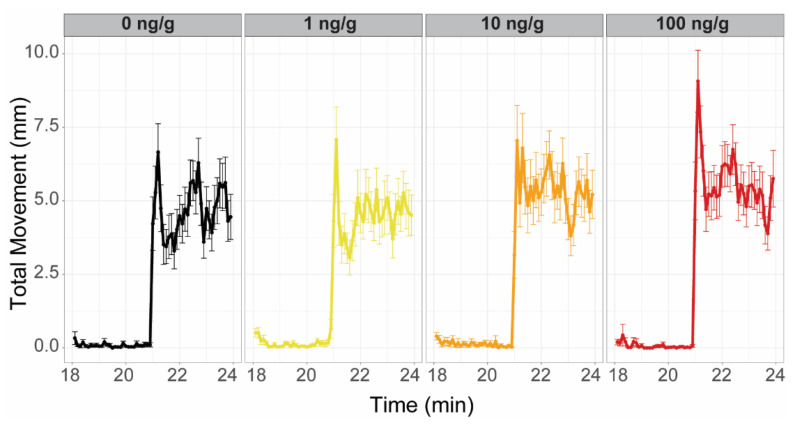
F1 Larval Photomotor Response. Plots represent one of the evaluated F1 zebrafish cohorts and are representative of both evaluated cohorts (120 hours post fertilization). Exposure groups are indicated on the top of each panel and by color (left to right, grey: 0 ng/g, yellow: 1 ng/g, orange: 10 ng/g, and red: 100 ng/g PFHxA added to the F0 diet). In each panel, the first 3 min were the final light period (lower total movement), while the last 3 min were the final dark period. The 10 ng/g group exhibited significant hyperactivity in the dark period relative to controls (*n* = 48).

To evaluate the effects of the F0 dietary exposure on the early development of subsequent generations, two cohorts from both the F1 and F2 generations were challenged with a larval photomotor response assay at 120 hpf. The F1 offspring of the F0 exposure groups were phenotypically normal but exhibited aberrant larval behavior. From both F1 cohorts, larvae from the 10 ng/g groups exhibited hyperactivity during the dark period of the larval photomotor response assay (Figure 6). Larvae from one F1 cohort also exhibited hyperactivity during the light period of the assay for the 10 and 100 ng/g groups. Consistent with the morphology data from the F1, no F2 cohorts exhibited abnormal morphology. F2 groups also did not exhibit abnormal larval photomotor behavior. Despite evidence of early biological perturbation in the F1 generation larvae, effects did not persist into the F2.

### 3.5. PFHxA Diet Fed to F0 Altered F1 but Not F2 Juvenile Shoaling Behavior

**Figure 7:**
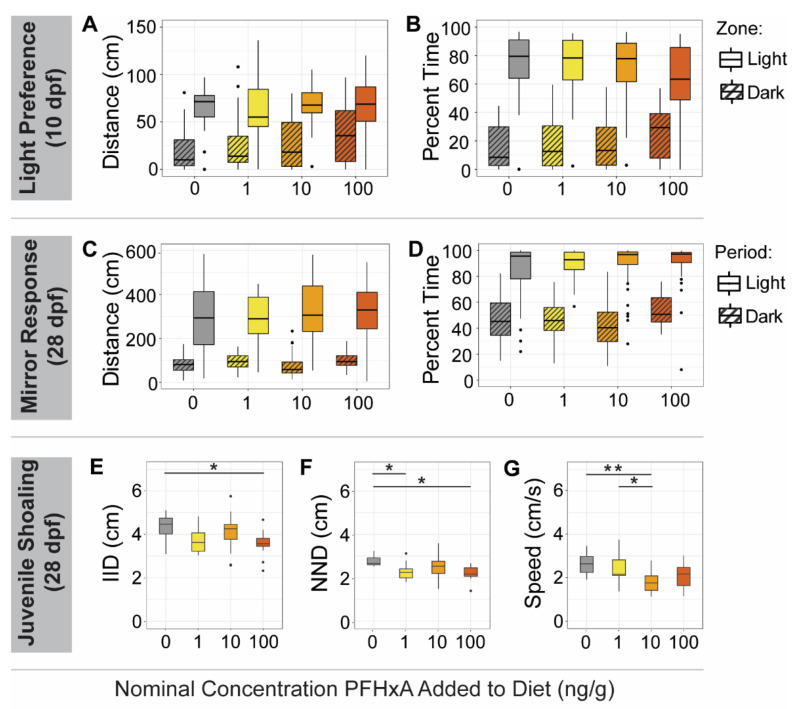
F1 Juvenile Behavior Assays. F1 zebrafish exposure groups are indicated on the x-axes and by color (left to right, grey: 0 ng/g, yellow: 1 ng/g, orange: 10 ng/g, and red: 100 ng/g PFHxA added to the F0 diet). During the light/dark preference assay at 10 dpf, no significant differences in total distance swam (**A**) or percent time spent (**B**) in the light or dark (striped boxes) assay zones were observed between exposure groups. In the mirror response assay at 28 dpf, no differences in distance traveled (**C**) or percent time spent (**D**) in the mirror zone during the light or dark periods (striped boxes) were noted. During the juvenile shoaling assay at 28 dpf, the average inter-individual distance (IID) of the 100 ng/g group was significantly lower than that of controls (**E**). Average nearest neighbor distance (NND) of the 1 and 100 ng/g groups were less than controls (**F**). Speed of the 10 ng/g group was significantly lower than that of controls (**G**). Significant differences between exposure groups are indicated on the plots (* *p* < 0.05; ** *p* < 0.005).

Further interrogating the effects of F0 juvenile dietary exposure on subsequent generations, F1 and F2 juveniles were evaluated for anxiety-like [46] and social [45] behaviors. To assess anxiety behavior, light/dark preference was measured at 10 dpf as percent time spent and distance traveled in a light or dark zone. None of the F1 or F2 exposure group juveniles exhibited abnormal light/dark preferences (Figure 7). Social behavior at 28 dpf were evaluated using a mirror response assay, in which percent time spent and distance traveled in the zone nearest a mirror were measured, and no aberrant social behavior was observed for either generation. An additional assessment of social perception (juvenile shoaling assay) was also conducted at 28 dpf by measuring inter-individual distance (IID), nearest neighbor distance (NND), and speed. Social perception of F1 offspring from all F0 exposure groups deviated from controls for at least one of the IID, NND, or speed endpoints (Figure 7). IID and NND were significantly decreased in the F1 100 ng/g group relative to controls, as well as NND in the F1 1 ng/g group, while the F1 10 ng/g group exhibited decreased speed. Aberrant social perception behavior did not persist into the F2 generation (Figure S7).

## 4. Discussion

The 42-day juvenile dietary exposure to 1, 10, or 100 ng/g PFHxA did not result in measurable whole-body concentration, apical morphometric effects, impaired fecundity, or abnormal social or associative learning behavior in adulthood for the directly exposed F0 or subsequent F1 or F2 generations. PFHxA exposure did elicit aberrant anxiety behavior in F0 adults, and effects persisted into the F1 adult offspring and transgenerationally into the F2. F0 exposure induced abnormal photomotor response in larval F1 offspring and aberrant shoaling behavior in juvenile F1s. The present findings largely concur with existing literature that PFHxA toxicity is subtle and manifests in sensitive, sublethal endpoints at environmentally relevant exposure concentrations. Evidence of multigenerational and transgenerational effects demonstrate that alternative short-chain PFAS, such as PFHxA, may not be devoid of toxicity, and behavior assays at various life stages will greatly contribute to safety assessments.

The lack of bioaccumulation observed in the present study concurred with other dietary exposure studies in various fish species that reported similar findings for PFHxA and other short-chain PFAS (i.e., hexafluoropropylene, also known as GenX), likely owing to rapid elimination from the organisms [47,48]. Rapid elimination of PFHxA has been reported in several laboratory mammalian species, with alpha phase serum elimination half-lives ranging from 0.3–2.4 h [27]. Longer serum elimination half-life estimates of 5.1 [27] to 14–49 [49] days have been reported for occupationally exposed ski wax technicians, still relatively rapid when compared to longer-chain PFAS. Lesser bioaccumulation may be a result of lower uptake of short-chain compounds by organic anion transporting polypeptides that would facilitate reabsorption [27]. However, lower bioaccumulative potential does not signify a lack of toxicity. Aqueous developmental exposures to PFHxA in zebrafish have induced abnormal larval behavior [28,29], illustrating the importance of investigating effects during the critical window of juvenile development, particularly via the highly relevant dietary route of exposure.

While select previous studies have employed gavage techniques to conduct dietary exposures in zebrafish [50], gavage was not feasible in the present study given the targeted juvenile life stage and the large sample size. Thus, measures were taken to ensure that PFHxA exposure occurred through the intended dietary route. PFHxA concentrations in the diets were analytically validated. Partial tank water exchanges occurred approximately 30 min after each feeding to maintain water quality and minimize aqueous exposure from any chemical that may have leached from the diet. While it was not possible to quantify the precise quantity of food consumed by the juvenile zebrafish, the lack of significant difference in growth between control and exposed populations indicated that similar amounts of food were consumed. The lack of measurable internal PFHxA concentration following exposures is a limitation of this study that should be considered, but rigorous maintenance of equivalent conditions across all exposure tanks provided confidence that observed effects were a result of the PFHxA dietary exposure.

The absence of apical morphometric effects in the F0 generation was consistent with other PFHxA dietary exposures in adult trout (*Oncorhynchus mykiss*) [47] and other PFAS mixture exposures (i.e., PFOS, PFOA, and GenX) in adult blue spot gobies (*Pseudogobius* sp.) [48]. In rodents, PFHxA effects on body weight and liver size have varied [27], and available rodent studies have not reported significant neurobehavioral effects from PFHxA. Lack of morphometric, fecundity, social behavior, and associative learning effects measured for the F0 population immediately following exposure and later in adulthood were evidence that the route of exposure and the resulting internal concentrations (not measurable after a 24 h fasting period) were not sufficient to induce effects. However, some abnormal anxiety behaviors observed using the predator, schooling, and startle response assays suggest that PFHxA dietary exposure subtly impacts juvenile development.

The PFHxA-contaminated diet fed to the F0 generation affected early development of the F1 generation, as evidenced by the abnormal larval behavior exhibited by the 10 ng/g exposure group lineage and juvenile shoaling behavior by all F1 groups. The present study used a comprehensive set of early developmental and juvenile behavior endpoints as indicators of biological perturbation. While longer-chain PFAS have been shown to transfer from mothers to embryos [51], this was not a consideration for PFHxA due to below-detection body burden. Instead, the F1 effects could be attributed to direct exposure to the germ line. At 17 dpf in zebrafish, perinuclear oocytes appear with gonad differentiation occurring between 21 and 40 dpf, at which point germ cells are present at multiple stages of oogenesis in female gonads, and maturation continues until 90 dpf [37]. Therefore, germ cells destined to become the F1 generation were present and exposed during the period that the F0 juveniles were fed a contaminated diet. Further investigation of PFHxA and other short-chain PFAS is warranted as the mechanisms of toxicity observed in the F1 generation are unclear. Effects may result from direct chemical exposure of the germ cells, altered biological processes within F0 fish to sufficiently affect germ cell development, or possibly epigenetic factors. Despite exposure effects early in development, once the F1 animals matured to adulthood, fecundity, social behavior, and associative learning were unaffected. However, abnormal anxiety behavior observed in the F0 generation was also noted in the F1 generation.

The present study also investigated F2 transgenerational effects, finding no abnormal larval or juvenile behavior, but aberrant adult anxiety behaviors. The lack of abnormal behavior early in F2 development suggests that such effects are not transgenerational. In contrast, aberrant anxiety behavior measured in adulthood persisted across the F0, F1, and F2 generations and is evidence of transgenerational effects. By leveraging the zebrafish model, the present study has rigorously interrogated the effects of juvenile dietary exposure to PFHxA, employing morphometric, fecundity, and behavior assessments across generations.

## 5. Conclusions

As manufacturing trends shift towards increased use of short-chain PFAS, careful safety assessment of these alternatives is imperative. Following the presently conducted 42-day dietary exposure to PFHxA in juvenile zebrafish, PFHxA was not measurable in whole body tissue samples and did not impact growth, fecundity, or social or learning behaviors. Subtle, multigenerational effects on anxiety-like behaviors were observed, demonstrating that extended exposure to low background concentrations during juvenile development induced persistent behavior effects, despite a lack of bioaccumulation. Findings warrant an additional assessment of alternative short-chain PFAS, in which behavior effects should be broadly considered before deeming them as safe.

## Figures and Tables

**Table 1:** Measured PFHxA concentrations in zebrafish diets.

Diet Granule Size	Nominal Concentration (ng/g)	Mean Measured Concentration (ng/g ± SD)
75-micron	0	0.50 ± 0.057
	1	1.0 ± 0.27
	10	5.6 ± 0.36
	100	260 ± 110
150-micron	0	0.24 ± 0.21
	1	1.1 ± 0.41
	10	5.1 ± 0.36
	100	310 ± 42

## Data Availability

The data presented in this study are available in the Supplementary Materials or upon request.

## Supplementary Materials

The following supporting information can be downloaded at: https://www.mdpi.com/article/10.3390/toxics10070372/s1, Section S1: Instrument Analysis by LC-MS/MS, and Extraction Method for Diet and Zebrafish Tissue; Section S2: Target PFHxA, Acronym, Neutral Molecular Formula, and Surrogate Standard for Analysis by LC-MS/MS; Section S3: Quantification and Quality Control; Section S4: Accuracy (Recovery %), Precision (Relative Standard Deviation %), and Limits of Detection (LOD) and Quantification (LOQ) for PFHxA in the Diet and Zebrafish Tissue Matrices [52]; Table S1: Analytical Validation of PFHxA Concentration in Diet; Table S2: Water Quality Standards; Table S3A: Developmental Toxicity Assessment Morphological Endpoints; Table S3B: Morphological Endpoints Additional Information; Table S4: Associative Behavior Assay Results; Figure S1: Adult Behavior Free Swim Assay– All Generations with Sex Plotted; Figure S2: Adult Behavior Predator Response Assay for F0, F1, and F2, Separated by Sex; Figure S3: Adult Behavior Startle Response for All Generations; Figure S4: Adult Behavior Shoaling Assay for F1 and F2; Figure S5: Adult Behavior Free Swim Assay for F1 and F2; Figure S6: Adult Behavior Predator and Schooling Response for F1 and F2; Figure S7: F2 Juvenile Behavior Assays; Data S1: Predator Response Assay Statistical Analysis-F0; Data S2: Predator Response Assay Statistical Analysis-F1; Data S3: Predator Response Assay Statistical Analysis-F2; Data S4: Schooling Response Assay Statistical Analysis-F0; Data S5: Schooling Response Assay Statistical Analysis-F1; Data S6: Schooling Response Assay Statistical Analysis-F2; Data S7: Startle Response Assay Statistical Analysis-F0; Data S8: Startle Response Assay Statistical Analysis-F1; Data S9: Startle Response Assay Statistical Analysis-F2.
