# Supplementary material for: Dietary Perfluorohexanoic Acid (PFHxA) Exposures in Juvenile Zebrafish Produce Subtle Behavioral Effects across Generations
Source: Toxics. 2022 Jul 4;10(7):372. doi: 10.3390/toxics10070372 (PMC9319656; doi:10.3390/toxics10070372)
Supplement: Supplementary file 1 [file toxics-10-00372-s001.zip › toxics-1780664-supplementary.pdf]

Supplemental Information

Contents:

Section S1. Instrument Analysis by LC-MS/MS, and Extraction Method for Diet and Zebrafish Tissue.

Section S2. Target PFHxA, Acronym, Neutral Molecular Formula, and Surrogate Standard for Analysis by LC-MS/MS.

Section S3. Quantification and Quality Control.

Section S4. Accuracy (Recovery %), Precision (Relative Standard Deviation %), and Limits of Detection (LOD) and Quantification (LOQ) for PFHxA in the Diet and Zebrafish Tissue Matrices.

Table S1. Analytical Validation of PFHxA Concentration in Diet.

Table S2. Water Quality Standards.

Table S3A. Developmental Toxicity Assessment Morphological Endpoints.

Table S3B. Morphological Endpoints Additional Information.

Table S4. Associative Behavior Assay Results.

Figure S1. Adult Behavior Free Swim Assay– All Generations with Sex Plotted.

Figure S2. Adult Behavior Predator Response Assay for F0, F1, and F2, Separated by Sex.

Figure S3. Adult Behavior Startle Response for All Generations.

Figure S4. Adult Behavior Shoaling Assay for F1 and F2.

Figure S5. Adult Behavior Free Swim Assay for F1 and F2.

Figure S6. Adult Behavior Predator and Schooling Response for F1 and F2.

Figure S7. F2 Juvenile Behavior Assays.

Data S1. Predator Response Assay Statistical Analysis- F0.

Data S2. Predator Response Assay Statistical Analysis- F1.

Data S3. Predator Response Assay Statistical Analysis- F2.

Data S4. Schooling Response Assay Statistical Analysis- F0.

Data S5. Schooling Response Assay Statistical Analysis- F1.

Data S6. Schooling Response Assay Statistical Analysis- F2.

Data S7. Startle Response Assay Statistical Analysis- F0.

Data S8. Startle Response Assay Statistical Analysis- F1.

Data S9. Startle Response Assay Statistical Analysis- F2.

Section S1. Instrument Analysis by LC-MS/MS, and Extraction Method for Diet and Zebrafish Tissue.

An Agilent 1100 series high-performance liquid chromatography system (Santa Clara, CA) and Waters Acquity triple quadrupole mass spectrometer (Milford, MA) were employed for separation and quantification of PFHxA in stock solution, diet, and homogenized subadult zebrafish tissue (60 days post fertilization). A C18 delay column (4.6 x 50 mm x 5 µm Zorbax Eclipse) was fitted between the LC pump and the autosampler. Chromatographic separation was achieved by using a C18 guard column (4.6 mm x 12.5 mm x 5 µm; P.N 820950-925) and Eclipse C18 analytical column (4.6x 75mm x 3.5µm; P.N. 959933-902). Mobile phase A was composed of 3% volume methanol in deionized water with 20 mM ammonium acetate and mobile phase B was methanol. The initial flow rate was 0.6 ml/min and mobile phase B was kept at 1% for 2 min and then increased to 60%, 85% and 99% at 3, 8.5 and 9 min, respectively, followed by a hold at 99% mobile phase B until 12.7 min. MS parameters can be found in Rericha et al. (2021) [1].

The PFHxA stock solution used to contaminate the zebrafish diet was previously validated by Rericha et al. (2021) [1].

For extraction of the diet samples, approximately 0.5 g of each sample was measured into a 15 mL polypropylene centrifuge tube and spiked with 0.9 ng surrogate standard. Samples were vigorously vortexed and let sit for 30 min. 3 mL acetonitrile was added, vortexed, and sonicated for 10 min at room temperature, followed by additional vortexing and centrifugation for 5 min at 4472 x g. Liquid was decanted into a new 15 mL tube, and the addition of acetonitrile and subsequent steps were repeated. 30 µL ethylene glycol was added, samples were evaporated until only the ethylene glycol remained, and then reconstituted with 3 mL methanol. Samples were once again vigorously vortexed, centrifuged for 5 min, then liquid was decanted into a new 15 mL tube. 3 mL methanol was added to a tube with an ENVI-Carb column (250 mg bed mass used due to the complexity of the diet matrix; preconditioned with 12 mL methanol), then the centrifugation, decanting, and addition of 3 mL to ENVI-carb column were repeated. Liquid was decanted, evaporated, and reconstituted to 150 µL methanol.

For extraction of zebrafish tissue, samples were homogenized in 200 µL acetonitrile with 0.5 mm stainless steel beads using a Bullet Blender (Next Advance) for at least 6 minutes at speed 8

or until samples appeared completely homogenized. Homogenate was transferred into a 15 mL polypropylene centrifuge tube, rinsing the original container three times with 1 mL acetonitrile. The extraction procedure was the same as described for the diet above, but a 100 mg dispersive ENVI-Carb column was used for the cleanup step.

Analysis of sample extracts entailed aliquoting 50 µL extract, diluting with 40 µL methanol, 50 µL NaCl solution (1.2 g/30 mL), and 10 µL (0.3 ng) internal standard (M2PFOA and M8PFOS) for injection into instrument. An injection volume of 100 µL was utilized for all diet and zebrafish tissue extracts.

Section S2. Target PFHxA, Acronym, Neutral Molecular Formula, and Surrogate Standard for Analysis by LC-MS/MS.

Chemical Name	Acronym	Neutral Molecular Formula[1]	Surrogate Standard
Perfluorohexanoic acid	PFHxA	C6HO2F11	M5PFHxA

1[M-H]- adduct were used for quantification

Section S3. Quantification and Quality Control.

Analytical-grade native and mass-labelled standards were purchased from Wellington labs (Guelph, Canada). The calibration curve for stock solutions ranged from 200 – 100,000 ng/L, and the two lowest points (200, 500 ng/L) of the calibration curve were used as quality control and required to be within 70%- 130%. Solvent blanks spiked with mass-labelled standard were used for checking the contamination and carryover. Concentrations of PFHxA were <LOD in all solvent blanks.

Section S4. Accuracy (recovery %), precision (relative standard deviation %), and limits of detection (LOD) and quantification (LOQ) for PFHxA in the diet and zebrafish tissue matrices.

LOD for PFHxA was calculated by using the method of Vial and Jardy (1999) [2], and LOQ LOD multiplied by 3.3.

Matrix	Accuracy %	Precision %	LOD	LOQ
Diet	96	13	0.06 ng/g	0.19 ng/g
Zebrafish tissue	110	15	0.07 ng/g	0.23 ng/g

Table S1. Analytical Validation of PFHxA Concentration in Diet.

Diet Granule Size	Nominal Concentration (ng/g)	Measured Concentration (ng/g)	Mean Measured Concentration (ng/g)
75-micron	0	0.54	0.50
		0.46	
		-	
	1	1.2	1.0
		1.2	
		0.73	
	10	5.5	5.6
		6	
		5.3	
	100	390	260
		190	
		200	
150-micron	0	0.0	0.24
		0.4	
		0.33	
	1	1.0	1.1
		1.6	
		0.81	
	10	5.4	5.1
		4.7	
		5.2	
	100	300	310
		280	
		360	

Table S2. Water Quality Standards.

Water Quality Parameter	Acceptable Range
Temperature	27-29 °C
pH	7-8
Ammonia	≤ 0.5 ppm
Nitrate	≤ 50 ppm
Nitrite	≤ 1 ppm

Table S3A. Developmental Toxicity Assessment Morphological Endpoints. Description of morphological endpoints and the timepoints at which they are assessed. See Table S3B for additional information on endpoint derivation and identification.

Endpoint	Definition
**MO24**	Mortality observed at 24 hpf
**DP24**	Delayed developmental by 24 hpf
**SM24**	Spontaneous movement at 24 hpf
**MORT**	Mortality occurring between 24 and 120 hpf
**CRAN**	Malformed, missing or smaller than normal eye, snout, and/or jaw at 120 hpf
**AXIS**	Curved or bent axis in either direction at 120 hpf
**EDEM**	Heart and/or yolk sac malformation, pericardial or yolk sac edema (fluid around the heart) at 120 hpf
**MUSC**	Lack of circulation, malformation or disorganized/missing somites, and improper swim bladder formation at 120 hpf
**LTRK**	Malformation of the lower trunk, including caudal fin region at 120 hpf
**BRN_**	Brain malformations or necrosis at 120 hpf
**SKIN**	Abnormal pigmentation at 120 hpf
**NC__**	Notochord malformation at 120 hpf
**TCHR**	Not responsive to touch at 120 hpf
**ANY.effect**	Combined incidence of any of the above-described morphological effects

Table S3B. Morphological Endpoints Additional Information. Original endpoints, as described with representative images on GitHub (https://github.com/Tanguay-Lab/Bioinformatic_and_Toxicological_Resources/tree/main/Files/Zebrafish_Phenotype_Atlas) have been collapsed into the current endpoints based on their correlated nature and frequency of co-occurrence. Current endpoints were assessed and reported for the present study.

**Current Endpoints**	**Abbreviation**	**Original Endpoints**		
**MO24**	**MO24**			
**DP24**	**DP24**			
**SM24**	**SM24**			
**Mortality**	**MORT**	MORT		
**Axis**	**AXIS**	AXIS		
**Brain**	**BRN_**	BRAIN	OTIC	PFIN
**Craniofacial**	**CRAN**	EYE	SNOUT	JAW
**Edema**	**EDEM**	YSE	PE	
**Lower Trunk**	**LTRK**	TRUNK	CFIN	
**Muscles**	**MUSC**	CIRC	SWIM	SOMITE
**Skin**	**SKIN**	PIG		
**Touch Response**	**TCHR**	TR		
**Notochord**	**NC__**	NC		

Table S4. Associative Behavior Assay Results.

Generation	Nominal Concentration PFHxA Added to Diet (ng/g)	Outcome	Female	Male	Percent	Average Critical Trial Number
**F0**	0	Not learner	10	3	21	-
		Learner	22	27	79	3.78
	1	Not learner	5	3	13	-
		Learner	25	29	87	5.24
	10	Not learner	7	2	15	-
		Learner	23	30	85	5.42
	100	Not learner	4	5	14	-
		Learner	27	27	86	4.94
**F1**	0	Not learner	5	4	14	-
		Learner	27	27	86	6.94
	1	Not learner	6	4	15	-
		Learner	26	29	85	5.73
	10	Not learner	5	4	14	-
		Learner	26	28	86	7.96
	100	Not learner	5	2	11	-
		Learner	27	30	89	6.04
**F2**	0	Not learner	13	11	36	-
		Learner	20	22	64	7.26
	1	Not learner	10	8	29	-
		Learner	21	23	71	7.36
	10	Not learner	11	16	42	-
		Learner	21	16	58	8.95
	100	Not learner	8	8	25	-
		Learner	24	24	75	7.23

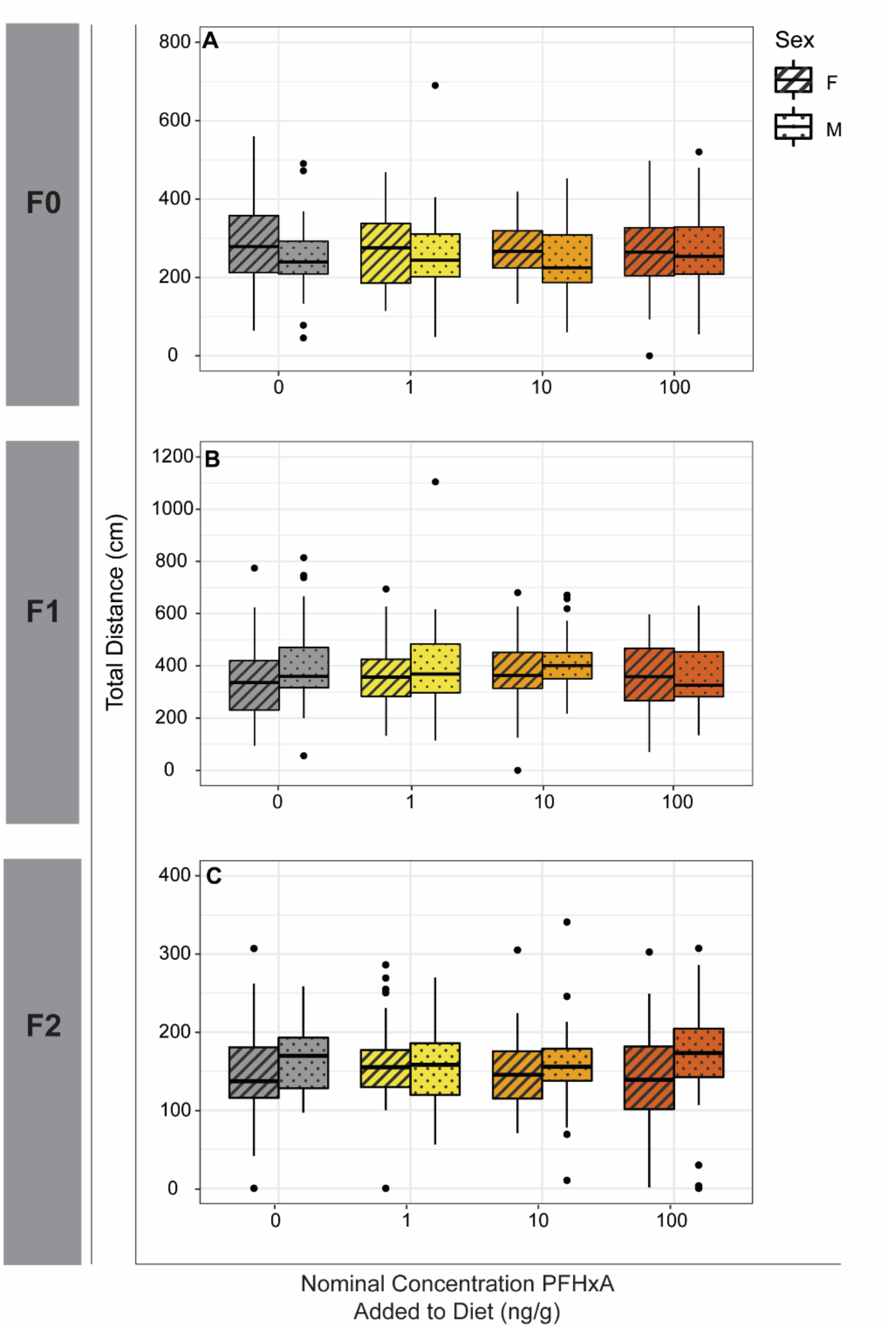

Figure S1. Adult Behavior Free Swim Assay– All Generations with Sex Plotted. Results from the Free Swim assay (n = 63-68, 32-35 males and 31-34 females) for all generations. Exposure groups are indicated on the x-axis and by color (grey: 0 ng/g, yellow: 1 ng/g, orange: 10 ng/g, and red: 100 ng/g PFHxA added to the F0 diet). Females are indicated by striped boxes, while males are indicated by dotted boxes. Within each generation, no significant difference in total distances between the sexes or the exposure groups was noted for the F0 (**A**), F1, (**B**), or the F2 (**C**; $p > 0.05$).

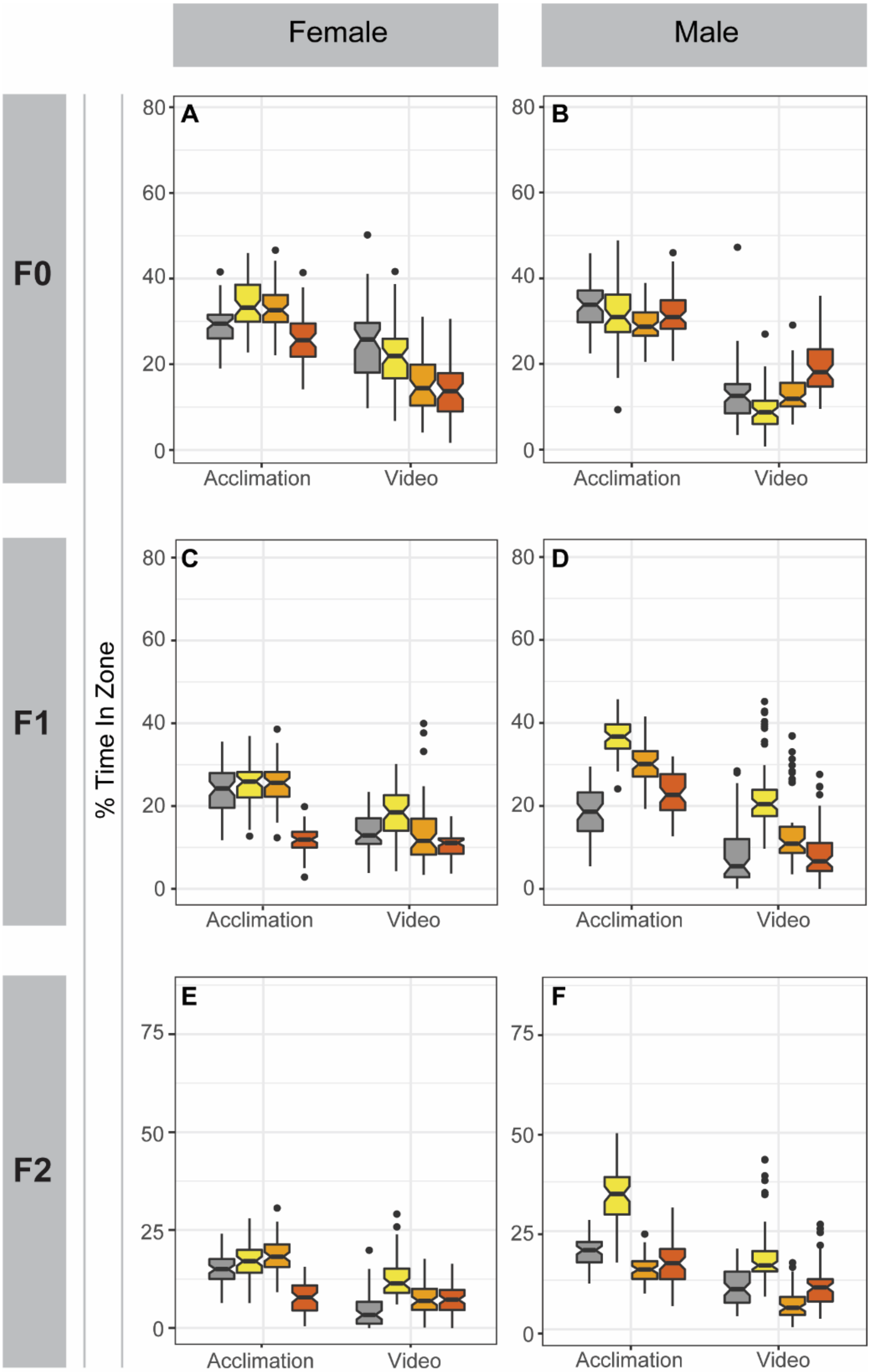

Figure S2. Adult Behavior Predator Response Assay for F0, F1, and F2, Separated by Sex. Plots show percent time (cumulative duration) spent in the near zone, closest to the video display before a predator video was displayed (Acclimation) and after (Video) for females (**A, C, E**) and males (**B, D, F**) from the F0, F1, and F2 generations (n = 56-68, 28-34 males and 27-34 females). Within each clustered set of boxplots, exposure groups are as follows left to right: Control (grey), 1 ng/g (yellow), 10 ng/g (orange), and 100 ng/g (red) PFHxA added to the F0 diet.

Significant differences determined by three-way ANOVA are not shown on plots but can be found in the supplementary Data S1-S3.

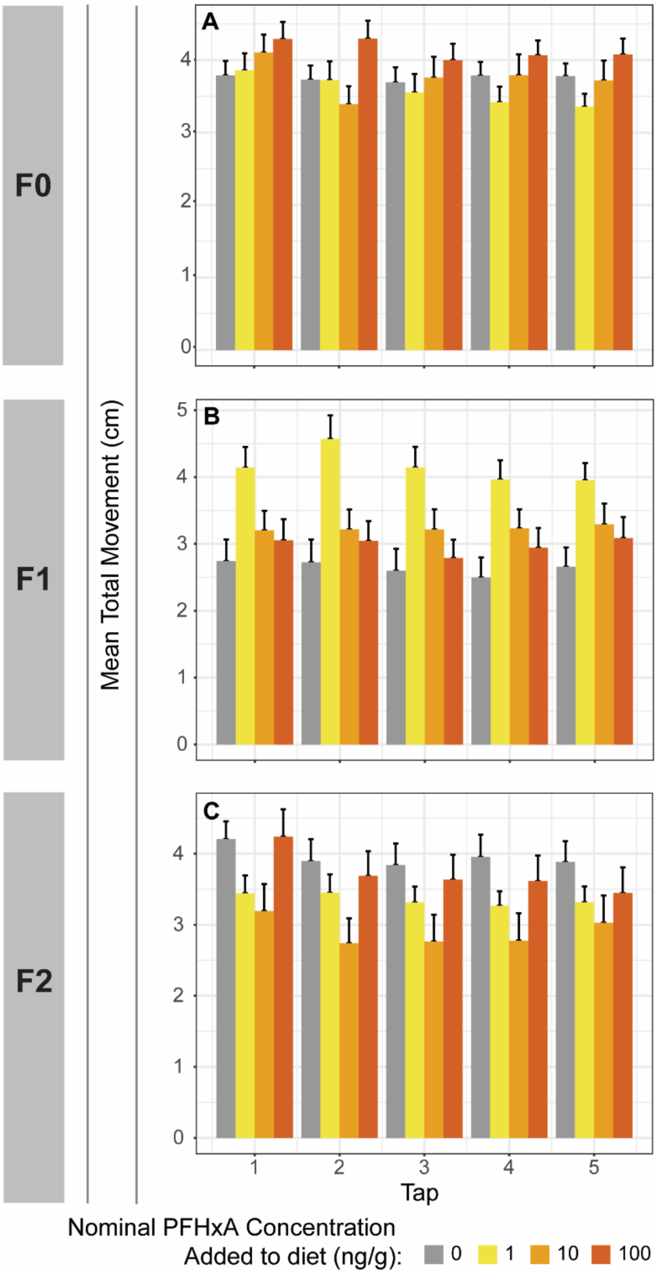

Figure S3. Adult Behavior Startle Response for All Generations.Exposure groups are indicated in the legend by color and within each cluster of bars (from left to right, grey: 0 ng/g, yellow: 1 ng/g, orange: 10 ng/g, and red: 100 ng/g PFHxA added to the diet). Startle response (n = 58-68, 29-35 males and 28-34 females) in average total movement (cm) was evaluated across a series of consecutive solenoid taps, indicated on the x-axis. In the F0 generation (**A**), the 100 ng/g group exhibited hyperactive startle response relative to controls (p = 0.035). For the F1 generation (**B**),

the 1 and 10 ng/g groups were hyperactive relative to controls ($p < 0.005$, $p = 0.010$, respectively). In the F2 generation (**C**), the 1 and 10 ng/g groups exhibited hypoactive startle response relative to controls ($p = 0.014$, $p < 0.005$, respectively). For the F1 and F2 generations, sex was a significant factor, and statistical analysis by repeated measure ANOVA can be found in the supplementary Data S7-S9.

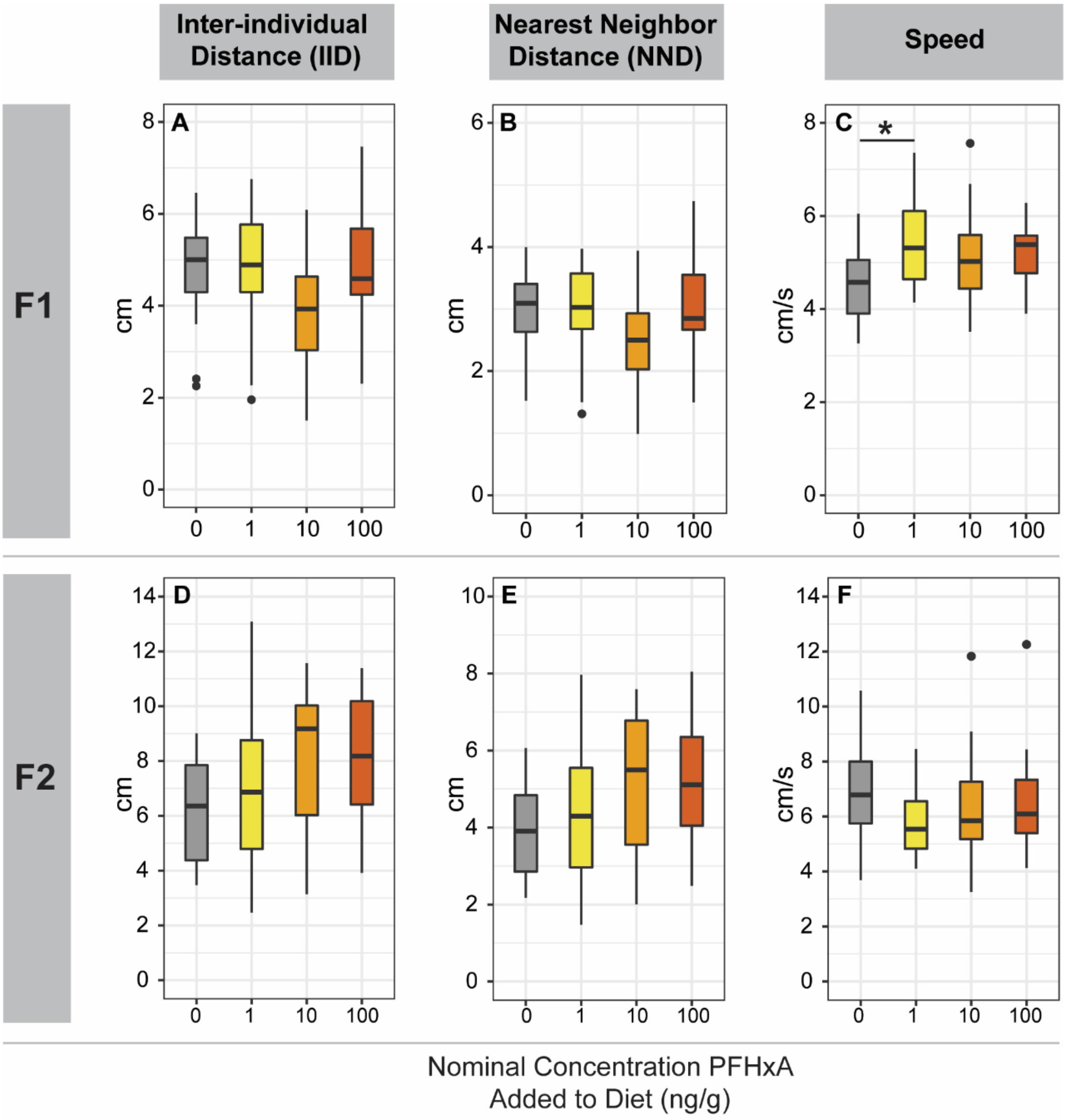

Figure S4. Adult Behavior Shoaling Assay for F1 and F2. Exposure groups are indicated on the x-axes and also by color (grey: 0 ng/g, yellow: 1 ng/g, orange: 10 ng/g, and red: 100 ng/g PFHxA added to the F0 diet). Assessing the behavior of 16 groups of 4 zebrafish, no significant exposure effects were observed for the F1 inter-individual distance (**A**) or nearest neighbor distance (**B**), but the 1 ng/g exposure group exhibited a higher speed than controls (**C**; $p = 0.024$). No significant effects were observed for the F2 generation (**D-F**) for any endpoints ($p > 0.05$).

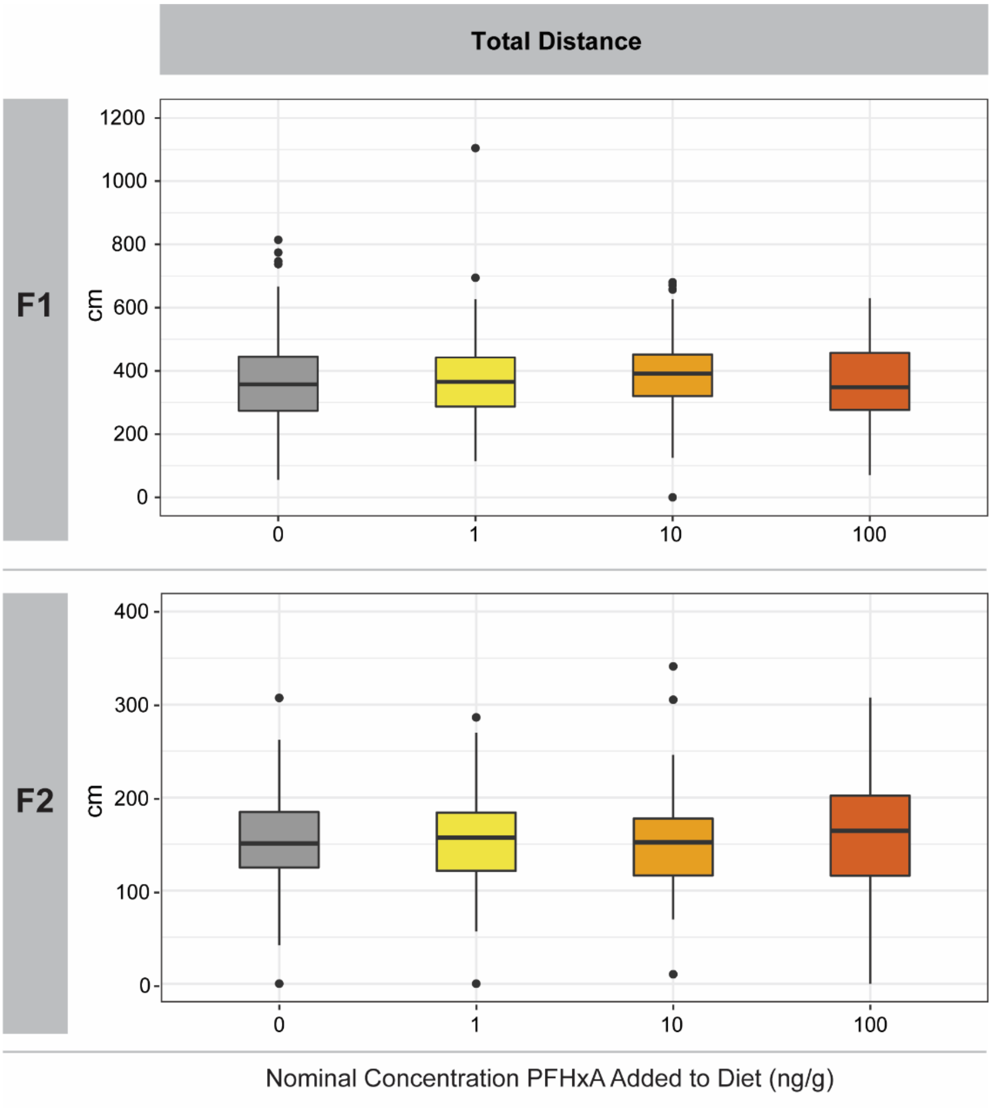

Figure S5. Adult Behavior Free Swim Assay for F1 and F2. Exposure groups are indicated on the x-axes and also by color (grey: 0 ng/g, yellow: 1 ng/g, orange: 10 ng/g, and red: 100 ng/g PFHxA added to the F0 diet). No significant exposure effects on average total distance swam ($n = 63\text{-}68$) were observed for the F1 (top) or the F2 (bottom) generation ($p > 0.05$).

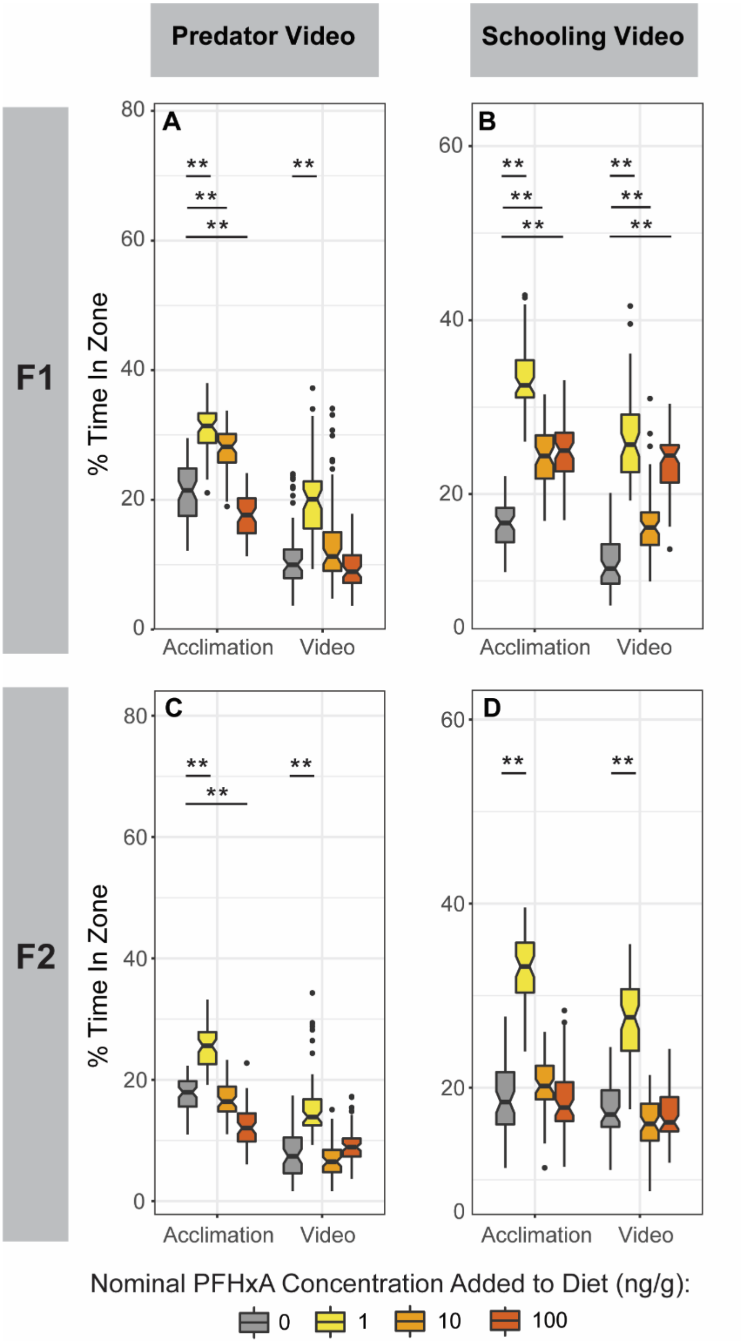

Figure S6. Adult Behavior Predator and Schooling Response for F1 and F2. Plots show percent time (cumulative duration) spent in the near zone, closest to the video display that showed a predator video (**A**,**C**) and a zebrafish schooling video (**B**,**D**). Exposure groups are indicated by color (grey: 0, yellow: 1, orange: 10, and red: 100 ng/g PFHxA added to the F0 diet). For both videos for the F1 (**A**,**B**) and F2 (**C**,**D**) generations (n = 56-64), the time spent in the near zone was significantly decreased for all exposure groups after the video was displayed (Video) compared to before the video was displayed (Acclimation) ($p < 0.005$), except for the F1 and F2 100 ng/g exposure groups and F2 controls in the schooling response. Significant differences between

groups within the Acclimation and Video periods are indicated on the plots (*p < 0.05; **p < 0.005).

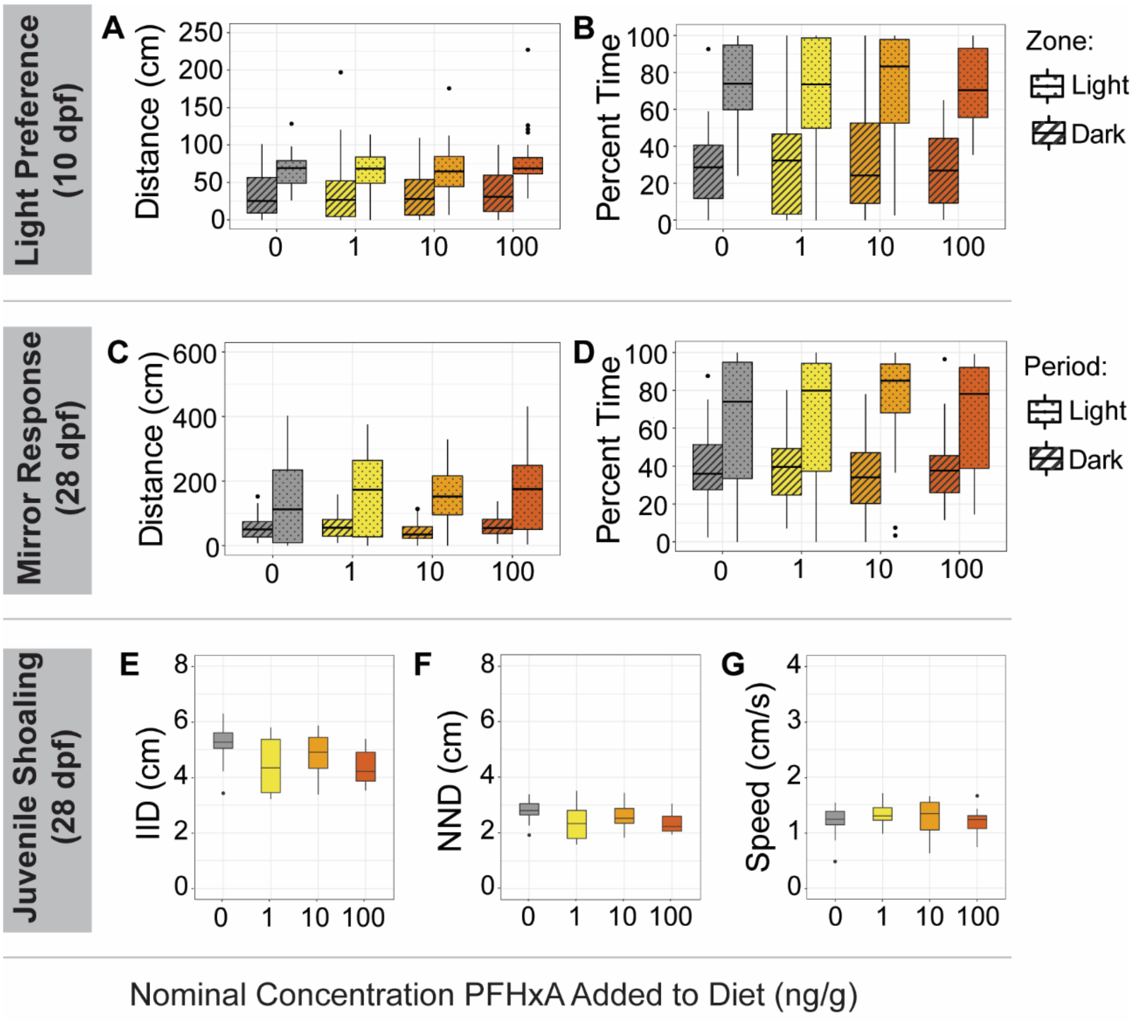

Figure S7. F2 Juvenile Behavior Assays. Results for the F2 generation challenged with juvenile behavior assays. Exposure groups (nominal concentration of PFHxA added to the diet fed to the F0 generation) are indicated on the x-axes and designated by color (grey: 0 ng/g, yellow: 1 ng/g, orange: 10 ng/g, and red: 100 ng/g PFHxA). There were no significant differences between exposure groups in the distance swam (**A**) or percent time spent (**B**) in the light (dotted boxes) or dark (striped boxes) zones in the 10 dpf light/dark preference assay. Distance swam (**C**) or percent time spent (**D**) in the mirror zone in the 28 dpf mirror response assay were also unaffected. Similarly, no significant differences were observed for inter-individual distance (IID; **E**), nearest neighbor distance (NND; **F**) or speed (**G**; p > 0.05).

Data S1. Predator Response Assay Statistical Analysis- F0.

	Df	Sum Sq	Mean Sq	F value	Pr(>F)	
Treatment	3	41019	13673	9.745	2.01E-06	***
status	1	1679530	1679530	1197.088	< 2e-16	***
Sex	1	43846	43846	31.251	2.29E-08	***
Treatment:status	3	49970	16657	11.872	9.11E-08	***
Treatment:Sex	3	195384	65128	46.42	< 2e-16	***
status:Sex	1	77533	77533	55.262	1.08E-13	***
Treatment:status:Sex	3	115900	38633	27.536	< 2e-16	***
Residuals	31780	44587761	1403			

Signif. codes: 0 '***' 0.001 '**' 0.01 '*' 0.05 '.' 0.1 ' ' 1

Tukey's multiple comparisons of means

95% family-wise confidence level

Fit: aov(formula = duration ~ Treatment * status * Sex, data)

$Treatment

	diff	lwr	upr	p adj
100 ng/g-Control	-2.540428	-4.05263648	-1.03E+00	0.0000937
1 ng/g-Control	-1.077934	-2.60E+00	0.4459992	0.265128
10 ng/g-Control	-2.782154	-4.31E+00	-1.2583211	0.0000162

$status

	diff	lwr	upr	p adj
Video-Acclimation	-14.53779	-15.36133	-13.71425	0

$Sex

	diff	lwr	upr	p adj
M-F	-2.347051	-3.170526	-1.523576	0

$`Treatment:status`

	diff	lwr	upr	p adj
100 ng/g:Acclimation-Control:Acclimation	-2.779354	-5.3237	-0.2350086	0.0209213
1 ng/g:Acclimation-Control:Acclimation	1.470473	-1.093692	4.0346386	0.6620879
10 ng/g:Acclimation-Control:Acclimation	-0.489729	-3.053552	2.0740944	0.9991189
Control:Video-Control:Acclimation	-12.323384	-14.837414	-9.8093543	0
100 ng/g:Video-100 ng/g:Acclimation	-11.850431	-14.383165	-9.3176963	0
1 ng/g:Video-1 ng/g:Acclimation	-17.330738	-19.902318	-14.7591586	0
10 ng/g:Video-10 ng/g:Acclimation	-16.831612	-19.402851	-14.2603735	0
100 ng/g:Video-Control:Video	-2.306401	-4.808679	0.1958768	0.096492
1 ng/g:Video-Control:Video	-3.536881	-6.058473	-1.0152894	0.0005619

10 ng/g:Video-Control:Video	-4.997957	-7.519549	-2.4763656	0.0000001

$`Treatment:Sex`

	diff	lwr	upr	p adj
100 ng/g:F-Control:F	-7.349567	-9.8630985	-4.83603552	0
1 ng/g:F-Control:F	0.7810541	-1.75161	3.31371823	0.9827226
10 ng/g:F-Control:F	-3.1516723	-5.7476365	-0.55570813	0.0057206
Control:M-Control:F	-4.1033289	-6.6170145	-1.58964334	0.0000207
100 ng/g:M-100 ng/g:F	5.5997366	3.0670725	8.13240074	0
1 ng/g:M-1 ng/g:F	-7.943034	-10.5145499	-5.37151799	0
10 ng/g:M-10 ng/g:F	-3.1235193	-5.7020412	-0.54499744	0.0058983
100 ng/g:M-Control:M	2.3534985	-0.1793185	4.88631549	0.0909174
1 ng/g:M-Control:M	-3.058651	-5.6114772	-0.5058247	0.0068414
10 ng/g:M-Control:M	-2.1718627	-4.667531	0.32380554	0.1424881

$`status:Sex`

	diff	lwr	upr	p adj
Video:F-Acclimation:F	-11.3811533	-12.9164019	-9.845905	0
Acclimation:M-Acclimation:F	0.8266358	-0.7127611	2.366033	0.5122131
Video:M-Video:F	-5.4163463	-6.9301985	-3.902494	0
Video:M-Acclimation:M	-17.6241354	-19.1421945	-16.106076	0

$`Treatment:status:Sex`

	diff	lwr	upr	p adj
100 ng/g:Acclimation:F-Control:Acclimation:F	-3.4377667	-7.4898001	0.61426683	0.2114188
1 ng/g:Acclimation:F-Control:Acclimation:F	5.0863486	1.003201	9.16949619	0.0021031
10 ng/g:Acclimation:F-Control:Acclimation:F	4.0422841	-0.1426381	8.22720631	0.0717893
Control:Video:F-Control:Acclimation:F	-3.9685643	-7.9872472	0.05011852	0.0572125
Control:Acclimation:M-Control:Acclimation:F	4.3909307	0.3388972	8.44296414	0.018912
100 ng/g:Video:F-100 ng/g:Acclimation:F	-11.6609746	-15.6796574	-7.64229174	0
100 ng/g:Acclimation:M-100 ng/g:Acclimation:F	5.7963516	1.713204	9.87949917	0.0001322
1 ng/g:Video:F-1 ng/g:Acclimation:F	-12.4333276	-16.5129779	-8.35367732	0
1 ng/g:Acclimation:M-1 ng/g:Acclimation:F	-2.8849289	-7.0309884	1.26113063	0.559844
10 ng/g:Video:F-10 ng/g:Acclimation:F	-18.1191477	-22.3973623	-13.84093309	0
10 ng/g:Acclimation:M-10 ng/g:Acclimation:F	-4.3390355	-8.4958391	-0.18223177	0.0304779
100 ng/g:Video:F-Control:Video:F	-11.1301769	-15.11523	-7.1451238	0
1 ng/g:Video:F-Control:Video:F	-3.3784147	-7.3935441	0.63671473	0.2234968
10 ng/g:Video:F-Control:Video:F	-10.1082992	-14.2240444	-5.99255407	0
Control:Video:M-Control:Video:F	-12.3211013	-16.306635	-8.33556762	0
100 ng/g:Video:M-100 ng/g:Video:F	5.4095864	1.394457	9.42471578	0.0004383
1 ng/g:Video:M-1 ng/g:Video:F	-12.8327784	-16.9092394	-8.75631739	0

10 ng/g:Video:M-10 ng/g:Video:F	-1.9478877	-6.0359791	2.14020376	0.9626837
100 ng/g:Acclimation:M-Control:Acclimation:M	-2.0323457	-6.1154933	2.05080186	0.9459573
1 ng/g:Acclimation:M-Control:Acclimation:M	-2.1895109	-6.304932	1.92591014	0.908635
10 ng/g:Acclimation:M-Control:Acclimation:M	-4.687682	-8.7106683	-0.66469572	0.0065734
Control:Video:M-Control:Acclimation:M	-20.6805963	-24.6997557	-16.66143689	0
100 ng/g:Video:M-100 ng/g:Acclimation:M	-12.0477398	-16.1273901	-7.9680895	0
1 ng/g:Video:M-1 ng/g:Acclimation:M	-22.3811771	-26.5240985	-18.23825574	0
10 ng/g:Video:M-10 ng/g:Acclimation:M	-15.7279999	-19.688857	-11.76714285	0
100 ng/g:Video:M-Control:Video:M	6.6005108	2.5849044	10.61611712	0.0000021
1 ng/g:Video:M-Control:Video:M	-3.8900918	-7.9374056	0.157222	0.0753854
10 ng/g:Video:M-Control:Video:M	0.2649144	-3.6920557	4.22188446	1

Data S2. Predator Response Assay Statistical Analysis- F1.

	Df	Sum Sq	Mean Sq	F value	Pr(>F)	
Treatment	3	563685	187895	154.791	< 2e-16	***
status	1	806158	806158	664.128	< 2e-16	***
Sex	1	38789	38789	31.955	1.59E-08	***
Treatment:status	3	34920	11640	9.589	2.52E-06	***
Treatment:Sex	3	174571	58190	47.938	< 2e-16	***
status:Sex	1	59435	59435	48.964	2.66E-12	***
Treatment:status:Sex	3	42536	14179	11.681	1.21E-07	***
Residuals	28539	34642321	1214			

Signif. codes: 0 '***' 0.001 '**' 0.01 '*' 0.05 '.' 0.1 ' ' 1

Tukey's multiple comparisons of means
95% family-wise confidence level

Fit: aov(formula = duration ~ Treatment * status * Sex, data)

$Treatment

	diff	lwr	upr	p adj
100 ng/g-Control	-2.725949	-4.24E+00	-1.213634	2.16E-05
1 ng/g-Control	9.17E+00	7.672275	10.66948	0.00E+00
10 ng/g-Control	4.37E+00	2.905629	5.841433	0.00E+00

$status

	diff	lwr	upr	p adj
Video-Acclimation	-10.62818	-11.4365	-9.819868	0

$Sex

	diff	lwr	upr	p adj
M-F	2.330322	1.522089	3.138555	0

$`Treatment:status`

	diff	lwr	upr	p adj
100 ng/g:Acclimation-Control:Acclimation	-3.6967609	-6.24E+00	-1.1521359	2.86E-04
1 ng/g:Acclimation-Control:Acclimation	9.57E+00	7.0456375	12.0885405	0.00E+00
10 ng/g:Acclimation-Control:Acclimation	6.49E+00	4.0205394	8.9598114	0.00E+00
Control:Video-Control:Acclimation	-9.7847597	-1.23E+01	-7.3156136	0.00E+00
100 ng/g:Video-100 ng/g:Acclimation	-7.8738371	-10.4507025	-5.2969716	0
1 ng/g:Video-1 ng/g:Acclimation	-10.5642363	-13.0960885	-8.0323841	0
10 ng/g:Video-10 ng/g:Acclimation	-13.9522644	-16.3818805	-11.5226483	0
100 ng/g:Video-Control:Video	-1.7858382	-4.2881975	0.716521	0.3744139
1 ng/g:Video-Control:Video	8.7876124	6.3078462	11.2673787	0
10 ng/g:Video-Control:Video	2.3226707	-0.1064475	4.7517888	0.0729287

$`Treatment:Sex`

	diff	lwr	upr	p adj
100 ng/g:F-Control:F	-7.6441336	-10.18167584	-5.1065913	0
1 ng/g:F-Control:F	2.5933017	0.12450426	5.0620992	0.0314622
10 ng/g:F-Control:F	0.6121297	-1.81745551	3.0417149	0.9948648
Control:M-Control:F	-5.2855111	-7.75465723	-2.8163649	0
100 ng/g:M-100 ng/g:F	4.4796308	1.90149822	7.0577633	0.0000039
1 ng/g:M-1 ng/g:F	8.2419617	5.70895477	10.7749686	0
10 ng/g:M-10 ng/g:F	2.3622897	-0.06729552	4.7918749	0.0636475
100 ng/g:M-Control:M	2.1210083	-0.38983405	4.6318507	0.170643
1 ng/g:M-Control:M	16.1207745	13.58742774	18.6541212	0
10 ng/g:M-Control:M	8.2599304	5.79078428	10.7290766	0

$`status:Sex`

	diff	lwr	upr	p adj
Video:F-Acclimation:F	-7.7677877	-9.259867	-6.2757086	0
Acclimation:M-Acclimation:F	5.2633407	3.75251	6.7741717	0
Video:M-Video:F	-0.5066607	-1.992563	0.9792412	0.817322
Video:M-Acclimation:M	-13.5377892	-15.04252	-12.0330583	0

$`Treatment:status:Sex`

	diff	lwr	upr	p adj
100 ng/g:Acclimation:F-Control:Acclimation:F	-12.19971945	-16.2904604	-8.1089785	0
1 ng/g:Acclimation:F-Control:Acclimation:F	1.113798943	-2.8661193	5.0937172	0.9998867
10 ng/g:Acclimation:F-Control:Acclimation:F	1.513414338	-2.4027882	5.4296169	0.9951071
Control:Video:F-Control:Acclimation:F	-10.07155974	-13.9862335	-6.156886	0
Control:Acclimation:M-Control:Acclimation:F	-5.581693362	-9.5621833	-1.6012035	0.0001772
100 ng/g:Video:F-100 ng/g:Acclimation:F	-1.10863006	-5.3032685	3.0860084	0.9999452
100 ng/g:Acclimation:M-100 ng/g:Acclimation:F	11.12296704	6.9664931	15.279441	0
1 ng/g:Video:F-1 ng/g:Acclimation:F	-7.161104033	-11.1404875	-3.1817206	0.0000001
1 ng/g:Acclimation:M-1 ng/g:Acclimation:F	11.82576255	7.742333	15.9091921	0
10 ng/g:Video:F-10 ng/g:Acclimation:F	-11.8471327	-15.7011255	-7.9931399	0
10 ng/g:Acclimation:M-10 ng/g:Acclimation:F	4.536317318	0.6201147	8.4525199	0.0072142
100 ng/g:Video:F-Control:Video:F	-3.236789768	-7.2599105	0.786331	0.2946338

1 ng/g:Video:F-Control:Video:F	4.024254652	0.1101247	7.9383846	0.0365286
10 ng/g:Video:F-Control:Video:F	-0.262158618	-4.1145979	3.5902806	1
Control:Video:M-Control:Video:F	-4.998786896	-8.9134606	-1.0841132	0.0013181
100 ng/g:Video:M-100 ng/g:Video:F	-1.943824349	-6.0310153	2.1433666	0.9632867
1 ng/g:Video:M-1 ng/g:Video:F	4.77591797	0.7599878	8.7918481	0.0047242
10 ng/g:Video:M-10 ng/g:Video:F	0.25873609	-3.5937032	4.1111754	1
100 ng/g:Acclimation:M-Control:Acclimation:M	4.504940948	0.4569274	8.5529545	0.013104
1 ng/g:Acclimation:M-Control:Acclimation:M	18.52125486	14.4372682	22.6052415	0
10 ng/g:Acclimation:M-Control:Acclimation:M	11.63142502	7.6509351	15.6119149	0
Control:Video:M-Control:Acclimation:M	-9.488653276	-13.4691432	-5.5081634	0
100 ng/g:Video:M-100 ng/g:Acclimation:M	-14.17542145	-18.223435	-10.1274079	0
1 ng/g:Video:M-1 ng/g:Acclimation:M	-14.21094861	-18.3300018	-10.0918954	0
10 ng/g:Video:M-10 ng/g:Acclimation:M	-16.12471393	-20.0393876	-12.2100402	0
100 ng/g:Video:M-Control:Video:M	-0.181827222	-4.1623171	3.7986627	1
1 ng/g:Video:M-Control:Video:M	13.79895952	9.7824994	17.8154197	0
10 ng/g:Video:M-Control:Video:M	4.995364368	1.0806907	8.9100381	0.0013357

Data S3. Predator Response Assay Statistical Analysis- F2.

	Df	Sum Sq	Mean Sq	F value	Pr(>F)	
Treatment	3.00E+00	458821	152940	167.96	< 2e-16	***
status	1	4.87E+05	487023	5.35E+02	< 2e-16	***
Sex	1.00E+00	203160	203160	2.23E+02	< 2e-16	***
Treatment:status	3.00E+00	64808	21603	2.37E+01	2.50E-15	***
Treatment:Sex	3	1.73E+05	57536	6.32E+01	< 2e-16	***
status:Sex	1.00E+00	20014	20014	2.20E+01	2.77E-06	***
Treatment:status:Sex	3	5.21E+04	17381	1.91E+01	2.32E-12	***
Residuals	29384	26756765	911			

Signif. codes: 0 ‘***’ 0.001 ‘**’ 0.01 ‘*’ 0.05 ‘.’ 0.1 ‘ ’ 1

Tukey’s multiple comparisons of means

95% family-wise confidence level

Fit: aov(formula = duration ~ Treatment * status * Sex, data)

$Treatment

	diff	lwr	upr	p adj
100 ng/g-Control	-1.8885621	-3.1701508	-0.6069735	0.0008819
1 ng/g-Control	8.0058982	6.7293217	9.2824748	0
10 ng/g-Control	-0.7842022	-2.0762529	0.5078486	0.4021345

$status

	diff	lwr	upr	p adj
Video-Acclimation	-8.141257	-8.83122	-7.451293	0

$Sex

	diff	lwr	upr	p adj
M-F	5.257377	4.567504	5.94725	0

$`Treatment:status`

	diff	lwr	upr	p adj
100 ng/g:Acclimation-Control:Acclimation	-5.3655316	-7.5218472	-3.2092161	0
1 ng/g:Acclimation-Control:Acclimation	7.9817395	5.833857	10.1296221	0
10 ng/g:Acclimation-Control:Acclimation	-0.7733242	-2.9472426	1.4005942	0.9614334
Control:Video-Control:Acclimation	-9.8791604	-12.0351862	-7.7231346	0
100 ng/g:Video-100 ng/g:Acclimation	-3.0392204	-5.1601865	-0.9182542	0.0003743
1 ng/g:Video-1 ng/g:Acclimation	-9.8316351	-11.9357008	-7.7275694	0
10 ng/g:Video-10 ng/g:Acclimation	-9.9005597	-12.0565854	-7.7445339	0
100 ng/g:Video-Control:Video	1.4744084	-0.6462631	3.5950799	0.4100642
1 ng/g:Video-Control:Video	8.0292648	5.9168869	10.1416427	0
10 ng/g:Video-Control:Video	-0.7947235	-2.9327069	1.3432599	0.9511671

$`Treatment:Sex`

	diff	lwr	upr	p adj
100 ng/g:F-Control:F	-2.1816165	-4.3198873	-0.04334575	0.0416574
1 ng/g:F-Control:F	5.032369	2.9105933	7.15414478	0
10 ng/g:F-Control:F	2.9624072	0.8066809	5.11813357	0.0008161
Control:M-Control:F	5.3686121	3.2128858	7.52433841	0
100 ng/g:M-100 ng/g:F	5.9547209	3.8340493	8.07539239	0
1 ng/g:M-1 ng/g:F	11.4981813	9.3941427	13.60221985	0
10 ng/g:M-10 ng/g:F	-2.1246067	-4.2803331	0.03111957	0.0567682
100 ng/g:M-Control:M	-1.5955077	-3.7337785	0.54276303	0.3151102
1 ng/g:M-Control:M	11.1619382	9.0236675	13.300209	0
10 ng/g:M-Control:M	-4.5308116	-6.6865379	-2.37508526	0

$`status:Sex`

	diff	lwr	upr	p adj
Video:F-Acclimation:F	-6.497632	-7.774006	-5.221258	0.00E+00
Acclimation:M-Acclimation:F	6.935244	5.645643	8.224845	0.00E+00
Video:M-Video:F	3.634522	2.366239	4.902806	0.00E+00
Video:M-Acclimation:m	-9.798353	-11.079948	-8.516759	0.00E+00

$`Treatment:status:Sex`

	diff	lwr	upr	p adj
100 ng/g:Acclimation:F-Control:Acclimation:F	-7.32037805	-10.76745826	-3.87329784	0
1 ng/g:Acclimation:F-Control:Acclimation:F	1.913145441	-1.50734337	5.33363425	0.8689352
10 ng/g:Acclimation:F-Control:Acclimation:F	3.144363842	-0.33085626	6.61958395	0.1301892
Control:Video:F-Control:Acclimation:F	-10.56140995	-14.00802694	-7.11479297	0
Control:Acclimation:M-Control:Acclimation:F	4.674991695	1.19977159	8.1502118	0.0004532
100 ng/g:Video:F-100 ng/g:Acclimation:F	-0.452370862	-3.84294156	2.93819983	1

100 ng/g:Acclimation:M-100 ng/g:Acclimation:F	8.584684527	5.16597583	12.00339322	0
1 ng/g:Video:F-1 ng/g:Acclimation:F	-4.425232414	-7.76240496	-1.08805987	0.0006156
1 ng/g:Acclimation:M-1 ng/g:Acclimation:F	17.08334409	13.69144921	20.47523897	0
10 ng/g:Video:F-10 ng/g:Acclimation:F	-10.91935735	-14.36597433	-7.47274036	0
10 ng/g:Acclimation:M-10 ng/g:Acclimation:F	-3.160384407	-6.63560451	0.3148357	0.1247815
100 ng/g:Video:F-Control:Video:F	2.788661042	-0.60143871	6.17876079	0.258299
1 ng/g:Video:F-Control:Video:F	8.049322982	4.68537507	11.41327089	0
10 ng/g:Video:F-Control:Video:F	2.786416448	-0.63135805	6.20419094	0.2726896
Control:Video:M-Control:Video:F	6.03949082	2.62171633	9.45726531	0.0000002
100 ng/g:Video:M-100 ng/g:Video:F	3.41098551	0.04878829	6.77318273	0.0425984
1 ng/g:Video:M-1 ng/g:Video:F	6.096138589	2.76031195	9.43196523	0
10 ng/g:Video:M-10 ng/g:Video:F	-1.122789016	-4.54056351	2.29498548	0.9992044
100 ng/g:Acclimation:M-Control:Acclimation:M	-3.410685218	-6.85776543	0.03639499	0.056052
1 ng/g:Acclimation:M-Control:Acclimation:M	14.32149783	10.87441762	17.76857804	0
10 ng/g:Acclimation:M-Control:Acclimation:M	-4.69101226	-8.16623237	-1.21579215	0.0004207
Control:Video:M-Control:Acclimation:M	-9.196910829	-12.64352781	-5.75029384	0
100 ng/g:Video:M-100 ng/g:Acclimation:M	-5.626069879	-9.01664058	-2.23549918	0.0000016
1 ng/g:Video:M-1 ng/g:Acclimation:M	-15.41243791	-18.80300861	-12.02186721	0
10 ng/g:Video:M-10 ng/g:Acclimation:M	-8.881761957	-12.32837894	-5.43514497	0
100 ng/g:Video:M-Control:Video:M	0.160155732	-3.22994402	3.55025548	1
1 ng/g:Video:M-Control:Video:M	8.105970751	4.715871	11.4960705	0
10 ng/g:Video:M-Control:Video:M	-4.375863388	-7.79363788	-0.95808889	0.0012519

Data S4. Schooling Response Assay Statistical Analysis- F0.

	Df	Sum Sq	Mean Sq	F value	Pr(>F)	
Treatment	3	95569	31856	19.707	9.29E-13	***
status	1	732543	732543	453.18	< 2e-16	***
Sex	1	946	946	0.585	0.444	
Treatment:status	3	183899	61300	37.922	< 2e-16	***
Treatment:Sex	3	745272	248424	153.685	< 2e-16	***
status:Sex	1	75294	75294	46.58	8.95E-12	***
Treatment:status:Sex	3	145680	48560	30.041	< 2e-16	***
Residuals	31900	51564769	1616			

Signif. codes: 0 '***' 0.001 '**' 0.01 '*' 0.05 '.' 0.1 ' ' 1

Tukey's multiple comparisons of means

95% family-wise confidence level

Fit: aov(formula = duration ~ Treatment * status * Sex, data)

$Treatment

	diff	lwr	upr	p adj
100 ng/g-Control	-4.3776239	-6.000786	-2.7544618	0
1 ng/g-Control	-2.952512	-4.5819092	-1.3231148	0.0000192
10 ng/g-Control	-4.0098354	-5.6454755	-2.3741953	0

$status

	diff	lwr	upr	p adj
Video-Acclimation	-9.583035	-10.46533	-8.700736	0

$Sex

	diff	lwr	upr	p adj
M-F	-0.3441144	-1.22639	0.538161	0.4446016

$`Treatment:status`

	diff	lwr	upr	p adj
100 ng/g:Acclimation-Control:Acclimation	-4.862157	-7.593187	-2.1311273	0.0000019
1 ng/g:Acclimation-Control:Acclimation	-2.5643161	-5.30593	0.177298	0.0865455
10 ng/g:Acclimation-Control:Acclimation	1.5898677	-1.162069	4.3418042	0.653421
Control:Video-Control:Acclimation	-6.9427727	-9.641262	-4.2442833	0
100 ng/g:Video-100 ng/g:Acclimation	-5.9880249	-8.706591	-3.2694585	0
1 ng/g:Video-1 ng/g:Acclimation	-7.703668	-10.442934	-4.9644022	0
10 ng/g:Video-10 ng/g:Acclimation	-17.9577591	-20.717655	-15.1978629	0
100 ng/g:Video-Control:Video	-3.9074092	-6.593284	-1.221534	0.0002776
1 ng/g:Video-Control:Video	-3.3252113	-6.021315	-0.6291078	0.00459
10 ng/g:Video-Control:Video	-9.4251187	-12.131725	-6.7185124	0

$`Treatment:Sex`

	diff	lwr	upr	p adj
100 ng/g:F-Control:F	-16.148205	-1.88E+01	-13.45008504	0
1 ng/g:F-Control:F	-3.1963028	-5.91E+00	-0.47764763	0.0087634
10 ng/g:F-Control:F	-7.2920349	-1.01E+01	-4.505439224	0
Control:M-Control:F	-8.0852954	-1.08E+01	-5.387175402	0
100 ng/g:M-100 ng/g:F	15.6910518	1.30E+01	18.40954284	0
1 ng/g:M-1 ng/g:F	-7.5987048	-1.03E+01	-4.859831303	0
10 ng/g:M-10 ng/g:F	-1.4131811	-4.18E+00	1.354532369	0.7814265
100 ng/g:M-Control:M	7.6281421	4.91E+00	10.3466332	0
1 ng/g:M-Control:M	-2.7097122	-5.43E+00	0.008778856	0.0514517
10 ng/g:M-Control:M	-0.6199206	-3.30E+00	2.058693574	0.9969787

$`status:Sex`

	diff	lwr	upr	p adj
Video:F-Acclimation:F	-6.466132	-8.114076	-4.818188	0.00E+00
Acclimation:M-Acclimation:F	2.777949	1.128633	4.427266	8.90E-05
Video:M-Video:F	-3.363463	-4.985414	-1.741512	6.00E-07
Video:M-Acclimation:M	-12.607544	-14.230891	-10.984198	0.00E+00

$`Treatment:status:Sex`

	diff	lwr	upr	p adj

100 ng/g:Acclimation:F-Control:Acclimation:F	-10.7825769	-15.1319167	-6.4332372	0
1 ng/g:Acclimation:F-Control:Acclimation:F	-0.0402764	-4.4230132	4.3424604	1
10 ng/g:Acclimation:F-Control:Acclimation:F	2.5134869	-1.9784919	7.0054657	0.8685609
Control:Video:F-Control:Acclimation:F	2.4294804	-1.8845732	6.743534	0.862631
Control:Acclimation:M-Control:Acclimation:F	1.4397337	-2.909606	5.7890735	0.9991303
100 ng/g:Video:F-100 ng/g:Acclimation:F	-8.126511	-12.4400531	-3.8129689	0
100 ng/g:Acclimation:M-100 ng/g:Acclimation:F	13.485313	9.1025762	17.8680497	0
1 ng/g:Video:F-1 ng/g:Acclimation:F	-3.7795408	-8.1585237	0.5994421	0.1874647
1 ng/g:Acclimation:M-1 ng/g:Acclimation:F	-3.6083878	-8.024269	0.8074934	0.2689734
10 ng/g:Video:F-10 ng/g:Acclimation:F	-16.8614551	-21.4535713	-12.2693388	0
10 ng/g:Acclimation:M-10 ng/g:Acclimation:F	-0.3784363	-4.8402335	4.0833609	1
100 ng/g:Video:F-Control:Video:F	-21.3385683	-25.616529	-17.0606076	0
1 ng/g:Video:F-Control:Video:F	-6.2492976	-10.5595375	-1.9390577	0.0000784
10 ng/g:Video:F-Control:Video:F	-16.7774485	-21.1956741	-12.359223	0
Control:Video:M-Control:Video:F	-17.3006402	-21.5786009	-13.0226794	0
100 ng/g:Video:M-100 ng/g:Video:F	17.8235264	13.5137984	22.1332543	0
1 ng/g:Video:M-1 ng/g:Video:F	-11.4565987	-15.7983696	-7.1148277	0
10 ng/g:Video:M-10 ng/g:Video:F	-2.4143892	-6.8024326	1.9736542	0.8831778
100 ng/g:Acclimation:M-Control:Acclimation:M	1.2630023	-3.1197345	5.6457391	0.9998366
1 ng/g:Acclimation:M-Control:Acclimation:M	-5.088398	-9.4711347	-0.7056612	0.0069596
10 ng/g:Acclimation:M-Control:Acclimation:M	0.6953168	-3.6228445	5.0134781	0.9999999
Control:Video:M-Control:Acclimation:M	-16.3108935	-20.6244356	-11.9973514	0
100 ng/g:Video:M-100 ng/g:Acclimation:M	-3.7882976	-8.1672805	0.5906853	0.1843609
1 ng/g:Video:M-1 ng/g:Acclimation:M	-11.6277516	-16.0067345	-7.2487688	0
10 ng/g:Video:M-10 ng/g:Acclimation:M	-18.897408	-23.1488815	-14.6459344	0
100 ng/g:Video:M-Control:Video:M	13.7855982	9.4758702	18.0953262	0
1 ng/g:Video:M-Control:Video:M	-0.4052561	-4.714984	3.9044719	1
10 ng/g:Video:M-Control:Video:M	-1.8911976	-6.1379794	2.3555842	0.9797647

Data S5. Schooling Response Assay Statistical Analysis- F1.

	Df	Sum Sq	Mean Sq	F value	Pr(>F)	
Treatment	3	900295	300098	218.66	< 2e-16	***
status	1	191755	191755	139.718	< 2e-16	***
Sex	1	1312	1312	0.956	0.328	
Treatment:status	3	41593	13864	10.102	1.20E-06	***
Treatment:Sex	3	234056	78019	56.847	< 2e-16	***
status:Sex	1	52672	52672	38.378	5.91E-10	***
Treatment:status:Sex	3	30053	10018	7.299	6.88E-05	***
Residuals	28544	39174916	1372			

Signif. codes: 0 '***' 0.001 '**' 0.01 '*' 0.05 '.' 0.1 ' ' 1

Tukey's multiple comparisons of means

95% family-wise confidence level

Fit: aov(formula = duration ~ Treatment * status * Sex, data)

$Treatment

	diff	lwr	upr	p adj
100 ng/g-Control	9.890311	8.276008	11.504613	0
1 ng/g-Control	15.459686	13.859844	17.059527	0
10 ng/g-Control	6.004046	4.442803	7.56529	0

$status

	diff	lwr	upr	p adj
Video-Acclimation	-5.183034	-6.042455	-4.323613	0

$Sex

	diff	lwr	upr	p adj
M-F	0.4284683	-0.4308636	1.2878	0.3284452

$`Treatment:status`

	diff	lwr	upr	p adj
100 ng/g:Acclimation-Control:Acclimation	8.455576303	5.7394591	11.171693	0
1 ng/g:Acclimation-Control:Acclimation	16.73367553	14.0418895	19.425462	0
10 ng/g:Acclimation-Control:Acclimation	7.752553573	5.125709	10.379398	0
Control:Video-Control:Acclimation	-4.311420002	-6.9583303	-1.66451	0.0000218
100 ng/g:Video-100 ng/g:Acclimation	-1.488991903	-4.2288044	1.250821	0.721375
1 ng/g:Video-1 ng/g:Acclimation	-6.817629752	-9.5097897	-4.12547	0
10 ng/g:Video-10 ng/g:Acclimation	-7.751106321	-10.3139662	-5.188246	0
100 ng/g:Video-Control:Video	11.2780044	8.6067848	13.949224	0
1 ng/g:Video-Control:Video	14.22746578	11.5801752	16.874756	0
10 ng/g:Video-Control:Video	4.312867254	1.7294446	6.89629	0.0000116

$`Treatment:Sex`

	diff	lwr	upr	p adj
100 ng/g:F-Control:F	4.6230271	1.92481682	7.3212374	0.0000057
1 ng/g:F-Control:F	10.7354849	8.11037207	13.3605977	0
10 ng/g:F-Control:F	7.2589721	4.67588548	9.8420587	0
Control:M-Control:F	-3.7636722	-6.41168639	-1.115658	0.0004379
100 ng/g:M-100 ng/g:F	6.6585453	3.9173646	9.3997259	0
1 ng/g:M-1 ng/g:F	6.0176915	3.32430376	8.7110793	0
10 ng/g:M-10 ng/g:F	-6.1480679	-8.71057183	-3.585564	0
100 ng/g:M-Control:M	15.0452446	12.35345861	17.7370306	0
1 ng/g:M-Control:M	20.5168486	17.80113516	23.232562	0
10 ng/g:M-Control:M	4.8745764	2.24663629	7.5025165	0.0000005

$`status:Sex`

	diff	lwr	upr	p adj
Video:F-Acclimation:F	-7.875672	-9.462117	-6.2892261	0
Acclimation:M-Acclimation:F	-2.332246	-3.938621	-0.7258709	0.0010985
Video:M-Video:F	3.098667	1.518846	4.6784889	0.0000028
Video:M-Acclimation:M	-2.444758	-4.044592	-0.8449248	0.0005013

$`Treatment:status:Sex`

	diff	lwr	upr	p adj
100 ng/g:Acclimation:F-Control:Acclimation:F	8.70E-01	-3.47963831	5.2198655	0.9999987
1 ng/g:Acclimation:F-Control:Acclimation:F	1.16E+01	7.33972955	15.8035542	0
10 ng/g:Acclimation:F-Control:Acclimation:F	9.45E+00	5.28890765	13.6172325	0
Control:Video:F-Control:Acclimation:F	-8.01E+00	-12.17424891	-3.8491755	0
Control:Acclimation:M-Control:Acclimation:F	-7.66E+00	-11.92419017	-3.3865274	0.0000001
100 ng/g:Video:F-100 ng/g:Acclimation:F	-6.29E-01	-5.08915931	3.8312964	1
100 ng/g:Acclimation:M-100 ng/g:Acclimation:F	7.50E+00	3.08376457	11.9218124	0.0000007
1 ng/g:Video:F-1 ng/g:Acclimation:F	-9.66E+00	-13.88795486	-5.4252676	0
1 ng/g:Acclimation:M-1 ng/g:Acclimation:F	3.03E+00	-1.31366591	7.3702892	0.555576
10 ng/g:Video:F-10 ng/g:Acclimation:F	-1.23E+01	-16.42495134	-8.2309896	0
10 ng/g:Acclimation:M-10 ng/g:Acclimation:F	-1.08E+01	-14.93219443	-6.6702318	0
100 ng/g:Video:F-Control:Video:F	8.25E+00	3.97504408	12.5307445	0
1 ng/g:Video:F-Control:Video:F	9.93E+00	5.7647843	14.0887014	0
10 ng/g:Video:F-Control:Video:F	5.14E+00	1.04148324	9.2321403	0.0018513
Control:Video:M-Control:Video:F	4.18E-04	-4.19784919	4.1986854	1
100 ng/g:Video:M-100 ng/g:Video:F	5.84E+00	1.49600498	10.1879593	0.0004606
1 ng/g:Video:M-1 ng/g:Video:F	8.91E+00	4.63885457	13.1792633	0
10 ng/g:Video:M-10 ng/g:Video:F	-1.65E+00	-5.71018075	2.4152111	0.9919222
100 ng/g:Acclimation:M-Control:Acclimation:M	1.60E+01	11.68886555	20.3676562	0
1 ng/g:Acclimation:M-Control:Acclimation:M	2.23E+01	17.87734382	26.6332808	0
10 ng/g:Acclimation:M-Control:Acclimation:M	6.31E+00	2.07074559	10.5436859	0.0000394
Control:Video:M-Control:Acclimation:M	-3.56E-01	-4.6596148	3.9477443	1
100 ng/g:Video:M-100 ng/g:Acclimation:M	-2.29E+00	-6.59341737	2.0139417	0.9086145
1 ng/g:Video:M-1 ng/g:Acclimation:M	-3.78E+00	-8.15572068	0.6039929	0.189049
10 ng/g:Video:M-10 ng/g:Acclimation:M	-3.17E+00	-7.27122304	0.9227387	0.3605186
100 ng/g:Video:M-Control:Video:M	1.41E+01	9.82679348	18.3621232	0
1 ng/g:Video:M-Control:Video:M	1.88E+01	14.52978328	23.1409841	0
10 ng/g:Video:M-Control:Video:M	3.49E+00	-0.67753221	7.6553498	0.2306849

Data S6. Schooling Response Assay Statistical Analysis- F2.

	Df	Sum Sq	Mean Sq	F value	Pr(>F)	
Treatment	3	854750	284917	215.51	< 2e-16	***
status	1	72363	72363	54.735	1.42E-13	***
Sex	1	26319	26319	19.908	8.16E-06	***
Treatment:status	3	25410	8470	6.407	0.000247	***
Treatment:Sex	3	686087	228696	172.985	< 2e-16	***
status:Sex	1	245	245	0.185	0.66674	
Treatment:status:Sex	3	58226	19409	14.681	1.50E-09	***
Residuals	29431	38909437	1322			

Signif. codes: 0 '***' 0.001 '**' 0.01 '*' 0.05 '.' 0.1 ' ' 1
Tukey's multiple comparisons of means
95% family-wise confidence level

Fit: aov(formula = duration ~ Treatment * status * Sex, data)

$Treatment

	diff	lwr	upr	p adj
100 ng/g-Control	-0.3022322	-1.846463	1.241999	0.9584333
1 ng/g-Control	12.2026617	10.670343	13.73498	0
10 ng/g-Control	0.1239089	-1.43691	1.684728	0.9970001

$status

	diff	lwr	upr	p adj
Video-Acclimation	-3.135553	-3.966229	-2.304876	0

$Sex

	diff	lwr	upr	p adj
M-F	-1.890781	-2.721366	-1.060197	8.10E-06

$`Treatment:status`

	diff	lwr	upr	p adj
100 ng/g:Acclimation-Control:Acclimation	-0.0955044	-2.6937245	2.50271567	1
1 ng/g:Acclimation-Control:Acclimation	14.450081	11.8719041	17.02825798	0
10 ng/g:Acclimation-Control:Acclimation	1.696351	-0.9253059	4.31800779	0.5084682
Control:Video-Control:Acclimation	-1.1242617	-3.7221326	1.47360925	0.8948043
100 ng/g:Video-100 ng/g:Acclimation	-1.5309393	-4.0865656	1.02468696	0.6093441
1 ng/g:Video-1 ng/g:Acclimation	-5.5454144	-8.0607921	-3.0300367	0
10 ng/g:Video-10 ng/g:Acclimation	-4.2455883	-6.8564969	-1.6346798	0.0000228
100 ng/g:Video-Control:Video	-0.5021821	-3.0574534	2.05308925	0.9989397
1 ng/g:Video-Control:Video	10.0289283	7.4933688	12.56448781	0
10 ng/g:Video-Control:Video	-1.4249757	-4.0119995	1.16204808	0.7071847

$`Treatment:Sex`

	diff	lwr	upr	p adj
100 ng/g:f-Control:f	-1.752754	-4.329231	0.8237233	0.4399547
1 ng/g:f-Control:f	18.388127	15.831525	20.9447288	0
10 ng/g:f-Control:f	10.842419	8.231503	13.4533352	0
Control:M-Control:F	5.75217	3.15466	8.3496798	0
100 ng/g:M-100 ng/g:F	8.653213	6.097942	11.2084846	0
1 ng/g:M-1 ng/g:F	-6.618761	-9.133789	-4.1037323	0
10 ng/g:M-10 ng/g:F	-15.525824	-18.13674	-12.914908	0
100 ng/g:M-Control:M	1.14829	-1.428188	3.7247668	0.8791617
1 ng/g:M-Control:M	6.017197	3.460595	8.5737984	0
10 ng/g:M-Control:M	-10.435574	-13.033084	-7.8380644	0

$`status:Sex`

	diff	lwr	upr	p adj
Video:F-Acclimation:F	-2.949522	-4.4912116	-1.4078323	0.0000053
Acclimation:M-Acclimation:F	-1.705571	-3.256924	-0.154218	0.0244704
Video:M-Video:F	-2.070592	-3.5987916	-0.5423923	0.0028125
Video:M-Acclimation:M	-3.314543	-4.8524907	-1.7765952	0.0000002

$`Treatment:status:Sex`

	diff	lwr	upr	p adj
100 ng/g:Acclimation:F-Control:Acclimation:F	-3.347436	-7.5009435	0.8060716	0.2917485
1 ng/g:Acclimation:F-Control:Acclimation:F	17.9026913	13.7812247	22.024158	0
10 ng/g:Acclimation:F-Control:Acclimation:F	13.2438174	9.0492635	17.4383714	0
Control:Video:F-Control:Acclimation:F	-2.8165919	-6.9695413	1.3363575	0.6059299
Control:Acclimation:M-Control:Acclimation:F	4.0303017	-0.1571126	8.217716	0.0743696
100 ng/g:Video:F-100 ng/g:Acclimation:F	0.3204875	-3.7649298	4.4059048	1
100 ng/g:Acclimation:M-100 ng/g:Acclimation:F	10.5341649	6.4148432	14.6534866	0
1 ng/g:Video:F-1 ng/g:Acclimation:F	-1.8616366	-5.8827126	2.1594394	0.9710764
1 ng/g:Acclimation:M-1 ng/g:Acclimation:F	-2.8749189	-6.9293653	1.1795276	0.5250712
10 ng/g:Video:F-10 ng/g:Acclimation:F	-7.6135509	-11.8086956	-3.4184062	0.0000001
10 ng/g:Acclimation:M-10 ng/g:Acclimation:F	-18.9870927	-23.1816467	-14.7925387	0
100 ng/g:Video:F-Control:Video:F	-0.2103566	-4.2952064	3.8744932	1
1 ng/g:Video:F-Control:Video:F	18.8576466	14.8043081	22.9109851	0
10 ng/g:Video:F-Control:Video:F	8.4468584	4.2933123	12.6004044	0
Control:Video:M-Control:Video:F	7.4211628	3.3029668	11.5393589	0.0000001
100 ng/g:Video:M-100 ng/g:Video:F	6.837512	2.7862829	10.888741	0.0000009
1 ng/g:Video:M-1 ng/g:Video:F	-10.2362737	-14.2236999	-6.2488475	0
10 ng/g:Video:M-10 ng/g:Video:F	-12.1295791	-16.2831252	-7.9760331	0
100 ng/g:Acclimation:M-Control:Acclimation:M	3.1564272	-0.9970803	7.3099348	0.3957741
1 ng/g:Acclimation:M-Control:Acclimation:M	10.9974708	6.8760041	15.1189374	0
10 ng/g:Acclimation:M-Control:Acclimation:M	-9.7735769	-13.9609912	-5.5861627	0
Control:Video:M-Control:Acclimation:M	0.5742693	-3.5786801	4.7272187	1
100 ng/g:Video:M-100 ng/g:Acclimation:M	-3.3761654	-7.4615827	0.7092518	0.2510069
1 ng/g:Video:M-1 ng/g:Acclimation:M	-9.2229915	-13.2440674	-5.2019155	0
10 ng/g:Video:M-10 ng/g:Acclimation:M	-0.7560374	-4.9089868	3.396912	0.9999996
100 ng/g:Video:M-Control:Video:M	-0.7940075	-4.8788573	3.2908423	0.9999991
1 ng/g:Video:M-Control:Video:M	1.20021	-2.8531285	5.2535486	0.9997715
10 ng/g:Video:M-Control:Video:M	-11.1038836	-15.2220797	-6.9856875	0

Data S7. Startle Response Assay Statistical Analysis- F0.

<u>Repeat measure ANOVA</u>

Error: Tap

	Df	Sum Sq	Mean Sq	F value	Pr(>F)
Treatment	2	7.179	3.59E+00	1.11E+00	0.475
Residuals	2	6.49E+00	3.245		

Error:
Tap:Treatment

	Df	Sum Sq	Mean Sq	F value	Pr(>F)	
Treatment	3	5.69E+01	1.90E+01	1.05E+01	0.00148	**
Sex	1	0.25	0.248	0.137	0.71829	
Residuals	11	19.9	1.809			

Signif. codes: 0 '***' 0.001 '**' 0.01 '*' 0.05 '.' 0.1 ' ' 1

Error: Within

	Df	Sum Sq	Mean Sq	F value	Pr(>F)
Sex	1	2	2.389	0.67	0.413
Residuals	1307	4659	3.564		

<u>Tukey's multiple comparisons of means</u>
95% family-wise confidence level

Fit: aov(formula = mean.distance ~ Treatment + Tap + Sex, data = (sum))

$Treatment

	diff	lwr	upr	p adj
100 ng/g-Control	0.3923129	0.0185067	0.7661191	0.0353862
1 ng/g-Control	-0.173236	-0.547615	0.2011426	0.6331063
10 ng/g-Control	-0.002461	-0.378004	0.3730816	0.9999983

$Sex

	diff	lwr	upr	p adj
M-F	0.0848896	-0.117905	0.2876843	0.4116851

Data S8. Startle Response Assay Statistical Analysis- F1.

<u>Repeat measure ANOVA</u>

Error: Tap

	Df	Sum Sq	Mean Sq	F value	Pr(>F)
Treatment	1	0.307	0.3065	0.128	0.745
Residuals	3	7.204	2.4015		

Error:Tap:Treatment

	Df	Sum Sq	Mean Sq	F value	Pr(>F)
Treatment	3	369	122.99	103.83	2.37E-08
Sex	1	0.1	0.15	0.124	0.732
Residuals	11	13	1.18		

Signif. Codes: 0 '***' 0.001 '**' 0.01 '*' 0.05 '.' 0.1 ' ' 1

Error: Within

	Df	Sum Sq	Mean Sq	F value	Pr(>F)
Sex	1	103	102.83	18.46	1.87E-05
Residuals	1178	6561	5.57		

Signif. Codes: 0 ‘***’ 0.001 ‘**’ 0.01 ‘*’ 0.05 ‘.’ 0.1 ‘ ’ 1

Tukey’s multiple comparisons of means
95% family-wise confidence level

Fit: aov(formula = mean.distance ~ Treatment + Tap + Sex, data = (sum))

$Treatment

	diff	lwr	upr	p adj
100 ng/g-Control	0.3378618	-0.1607531	0.8364767	0.301743
1 ng/g-Control	1.5090172	1.0130805	2.0049538	0
10 ng/g-Control	0.5870195	0.103146	1.0708929	0.0099611

$Sex

	diff	lwr	upr	p adj
M-F	0.5853587	0.3190141	0.8517034	1.75E-05

Data S9. Startle Response Assay Statistical Analysis- F2.

Repeat measure ANOVA

Error: Tap

	Df	Sum Sq	Mean Sq	F value	Pr(>F)
Residuals	4	25.07	6.268		

Error:
Tap:Treatment

	Df	Sum Sq	Mean Sq	F value	Pr(>F)	
Treatment	3	190.17	63.39	55.18	2.71E-07	***
Residuals	12	13.78	1.15			

Signif. Codes: 0 ‘***’ 0.001 ‘**’ 0.01 ‘*’ 0.05 ‘.’ 0.1 ‘ ’ 1

Error: Within

	Df	Sum Sq	Mean Sq	F value	Pr(>F)	
Sex	1	211	211.1	34.59	5.27E-09	***
Residuals	1204	7346	6.1			

Signif. Codes: 0 ‘***’ 0.001 ‘**’ 0.01 ‘*’ 0.05 ‘.’ 0.1 ‘ ’ 1

Tukey’s multiple comparisons of means
95% family-wise confidence level

Fit: aov(formula = mean.distance ~ Treatment + Tap + Sex, data =
(sum))

$Treatment

	diff	lwr	upr	p adj
100 ng/g-Control	-0.2306936	-0.7432802	0.28189295	0.6536217
1 ng/g-Control	-0.5967469	-1.1053793	-0.0881146	0.0138094
10 ng/g-Control	-1.0551503	-1.5741064	-0.5361942	0.0000012

$Sex

	diff	lwr	upr	p adj
M-F	0.8301344	0.5543181	1.105951	0

References

1.Rericha, Y.; Cao, D.; Truong, L.; Simonich, M.; Field, J.A.; Tanguay, R.L. Behavior effects of structurally diverse per- and polyfluoroalkyl substances in zebrafish. *Chem. Res. Toxicol.* **2021**, *34*, 1409–1416.

2.Vial J.; Jardy A. "Experimental Comparison of the Different Approaches To Estimate LOD and LOQ of an HPLC Method," *Analytical Chemistry*, **1999**. *71*, 2672–2677.
