# Supplementary material for: Dietary Perfluorohexanoic Acid (PFHxA) Exposures in Juvenile Zebrafish Produce Subtle Behavioral Effects across Generations
Source: Toxics. 2022 Jul 4;10(7):372. doi: 10.3390/toxics10070372 (PMC9319656; doi:10.3390/toxics10070372)
Supplement: Supplementary file 1 [file toxics-10-00372-s001.zip › Raw Data.zip!/toxics-1780664-Raw Data Guide Supporting Information.pdf]

Raw Data Guide

Folder and File Layout:

1) **60 dpf Morphometrics:**
    a. 60 dpf Length and Weight- F0
    b. 60 dpf Organ Weights- F0
2) **Adult Behavior:**
    a. **Free Swim**
        i. Free Swim- F0
        ii. Free Swim- F1
        iii. Free Swim- F2
    b. **Predator Response**
        i. Predator Response- F0
        ii. Predator Response- F1
        iii. Predator Response- F2
    c. **Schooling Response**
        i. Schooling Response- F0
        ii. Schooling Response- F1
        iii. Schooling Response- F2
    d. **Shoaling**
        i. Adult Shoaling- F0
        ii. Adult Shoaling- F1
        iii. Adult Shoaling- F2
    e. **Startle Response**
        i. Startle Response- F0
        ii. Startle Response- F1
        iii. Startle Response- F2
3) **Developmental Toxicity**
    a. **Larval Photomotor Response**
        i. Larval Photomotor Response- F1
        ii. Larval Photomotor Response- F2
    b. **Morphology**
        i. Morphology- F1
        ii. Morphology- F2
4) **Fecundity**
    a. Fecundity- F0
    b. Fecundity- F1
    c. Fecundity- F2
5) **Juvenile Behavior**
    a. **Juvenile Shoaling**
        i. Juvenile Shoaling- F1

            ii. Juvenile Shoaling- F2
   b. **Light Dark Preference**
      i. Light Dark Preference- F1
      ii. Light Dark Preference- F2
   c. **Mirror Response**
      i. Mirror Response- F1
      ii. Mirror Response- F2

<u>60 dpf Morphometrics Data Explanation</u>

60 dpf Length and Weight- F0:

F0 zebrafish were measured for length and weight at 60 dpf, immediately following the 42-day dietary exposure. K-factor was calculated as a measure of body length to weight relationship.

Column 1 contains the exposure group (nominal ng/g PFHxA in F0 diet), and Columns 2-4 contain length (mm), weight (g), and K-factor data.

60 dpf Organ Weights- F0:

F0 zebrafish were dissected, and brains and livers were pooled and weighed.

Column 1 contains the exposure group (nominal ng/g PFHxA in F0 diet). Columns 2-3 contain the type of organ and the number of pooled organs. Columns 4-5 contain the measured pooled organ weight (g) and the average organ weight (mg), respectively.

<u>Adult Behavior Data Explanation:</u>

Free Swim:

Adult zebrafish from the F0, F1, and F2 generations were allowed uninterrupted free swim (1 fish per tank) for 30 min without any external stimulus, and swimming distance was measured.

The first two columns contain metadata including filename and tank location. Each unique combination of filename and tank location represents an individual fish. Columns 3-6 contain additional metadata: the exposure group (nominal ng/g PFHxA in F0 diet), sex of the fish, timestamp (each representing 60 s), and assay phase. Data from the acclimation phase was not analyzed in the present study; all analysis was conducted on data from the effect phase. Column 7 contains the measured endpoint of the mean distance swam (cm).

Predator and Schooling Response:

Adult zebrafish from the F0, F1, and F2 generations were placed into an array of tanks (1 fish per tank) in which each tank displayed a video projection on one side. For the purposes of analysis, each tank was divided into three zones of equal width: close (i.e., closest to the video projection), middle, and far (i.e., farthest from the video projection). Fish underwent an acclimation period (20 min) followed by a period during which a predator video was displayed (5 min), a second acclimation (5 min), and a period during which a schooling zebrafish video was displayed (5 min). The data provided represents 1 min prior to and 1 min after the video display.

The first two columns contain metadata including filename and tank location. Each unique combination of filename and tank location represents an individual fish. Columns 3-6 contain additional metadata: the exposure group (nominal ng/g PFHxA in F0 diet), sex of the fish, timestamp (each representing 1 s), and assay phase. In the present study, the 1 minute before the video was displayed (Acclimation) and the 1 minute after the video was displayed (Video) were

analyzed. Columns 7-9 contain the measured endpoints of cumulative percent time spent in either the close, middle, or far assay zones.

Shoaling:

Adult zebrafish from the F0, F1, and F2 generations were placed in tanks as groups of 4 fish (2 males and 2 females), allowed to swim without any external stimulus for 30 min, and inter-individual distance (IID), nearest neighbor distance (NND), and speed were measured.

The first two columns contain metadata including filename and tank location. Each unique combination of filename and tank location represents one group of 4 fish. Columns 3-5 contain additional metadata: the exposure group (nominal ng/g PFHxA in F0 diet), timestamp (each representing 60 s), and assay phase. Data from the acclimation phase was not analyzed in the present study; all analysis was conducted on data from the effect phase. Columns 6-8 contain the measured endpoints IID (cm), NND (cm), and speed (cm/s).

Startle Response:

Adult zebrafish from the F0, F1, and F2 generations were assessed for startle response through the generation of 10 consecutive taps (every 20 s) underneath the tanks (1 fish per tank).

The first two columns contain metadata including filename and tank location. Each unique combination of filename and tank location represents an individual fish. Columns 3-6 contain additional metadata: the exposure group (nominal ng/g PFHxA in F0 diet), sex of the fish, timestamp (each representing 1 s), and status (tap number). Column 7 contains the measured endpoints of mean distance swam (cm).

<u>Developmental Toxicity Data Explanation:</u>

Larval Photomotor Response:

Developmental zebrafish from the F1 and F2 generations were assessed for normal behavior at 120 hours post-fertilization (hpf).

Each row represents one larvae over time. The first 3 columns consist of metadata, including exposure group (nominal ng/g PFHxA in F0 diet), an id and date for each 96-well plate, and well location of each animal within a 96-well plate. The following columns contain time series data, with 240 datapoints (one every 6 s).

Note: Removal of dead or malformed fish is highly recommended, but all data provided is uncensored. The light (L) and dark (D) cycles in the assay are as follows: L1: T61-89; D1: T90-119; L2: T120-149; D2: T150-179; L3: T180-209; D3: T210-239. The first 2 cycles were treated as acclimation and the third cycle was analyzed in the current study.

Morphology:

Developmental zebrafish from the F1 and F2 generations were assessed for normal development at 24 and 120 hpf.

This is animal/well level data where each row represents an animal. There are 18 total columns. The first 4 columns are metadata including exposure group (nominal ng/g PFHxA in F0 diet), an id and date for each 96-well plate, and well location of each animal within a 96-well plate. These are followed by 13 columns for mortality and morphology endpoints at either 24 or 120 hpf. Below in Table A is a description of each endpoint. The current endpoints were collapsed from original endpoints, detailed in Table B, based on correlated nature and frequency of co-occurrence (more information and representative images available at https://github.com/Tanguay-

Lab/Bioinformatic_and_Toxicological_Resources/tree/main/Files/Zebrafish_Phenotype_Atlas). The last column is a well quality control column ("DNC").

Note: If an embryo is dead at 24 hpf (noted as "MO24", all subsequent endpoints will be "NA" as only viable embryos are evaluated). Therefore, the MORT column represents only those that are dead at 120 hpf and does not take into consideration those dead at 24 hpf. To consider total mortality, if MO24 == 1, MORT should be 1 also. Additionally, the endpoint "DNC_" is not a morphological endpoint, but well quality control. Therefore, if DNC == 1, that well should be discarded from the analysis completely.

Table A. Mortality and morphology endpoint descriptors.

Column Names	Definition
**MO24**	Mortality observed at 24 hpf
**DP24**	Delayed developmental by 24 hpf
**SM24**	Spontaneous movement at 24 hpf
**MORT**	Mortality occurring between 24 and 120 hpf
**CRAN**	Malformed, missing or smaller than normal the eye, snout, and/or jaw at 120 hpf
**AXIS**	Curved or bent axis in either direction at 120 hpf
**EDEM**	Heart and/or yolk sac malformation, pericardial or yolk sac edema (fluid around the heart) at 120 hpf
**MUSC**	Lack of circulation, malformation or disorganized/ missing somites, and improper swim bladder formation at 120 hpf
**LTRK**	Malformation of the lower trunk, including the caudal fin region at 120 hpf
**BRN_**	Brain malformations or necrosis at 120 hpf
**SKIN**	Abnormal pigmentation at 120 hpf
**NC__**	Notochord malformation at 120 hpf
**TCHR**	Not responsive to touch at 120 hpf
**DNC_**	Well quality control: 1 = poor well quality

Table B. Original endpoints collapsed into current endpoints.

Current Endpoints	Abbreviation	Original Endpoints		
**MO24**	**MO24**			
**DP24**	**DP24**			
**SM24**	**SM24**			
**Mortality**	**MORT**	MORT		
**Axis**	**AXIS**	AXIS		
**Brain**	**BRN_**	BRAIN	OTIC	PFIN
**Craniofacial**	**CRAN**	EYE	SNOUT	JAW
**Edema**	**EDEM**	YSE	PE	
**Lower Trunk**	**LTRK**	TRUNK	CFIN	

**Muscles**	**MUSC**	CIRC	SWIM	SOMITE
**Skin**	**SKIN**	PIG		
**Touch Response**	**TCHR**	TR		
**Notochord**	**NC__**	NC		

<u>Fecundity Data Explanation:</u>

Adult zebrafish from the F0, F1, and F2 generations were spawned in groups of 5 (3 males and 2 females) for 5-6 consecutive spawning events approximately 10 days apart to assess fecundity.

Each row represents one spawning group. Columns 1-3 contain metadata, including date of spawn, exposure group (nominal ng/g PFHxA in F0 diet), and an id for the spawning set within each exposure group. Columns 4-6 consist of counts of fertilized, unfertilized, and necrotic embryos.

<u>Juvenile Behavior Data Explanation:</u>

Juvenile Shoaling

Juvenile zebrafish from the F1 and F2 generations were placed in arenas as groups of 4 fish, allowed to swim without any external stimulus for 7 min, and inter-individual distance (IID), nearest neighbor distance (NND), and speed were measured.

The first two columns contain metadata including filename and exposure group (nominal ng/g PFHxA in F0 diet). Each unique combination of filename and exposure group represents one group of 4 fish. Column 3 contains additional timestamp metadata (each representing 60 s). Columns 4-6 contain the measured endpoints IID (cm), NND (cm), and speed (cm/s).

Light Dark Preference

Juvenile zebrafish from the F1 and F2 generations were placed individually into arenas containing a dark and light zone, each of equal area. Fish were allowed to acclimate without external lighting of the arena for 6 min and then swim for 6 min with lighting while time spent and swimming distance were measured to assess zone preference.

The first two columns contain metadata including filename and well location. Each unique combination of filename and location represents an individual fish. Columns 3-6 contain additional metadata, including exposure group (nominal ng/g PFHxA in F0 diet), zone (dark or light), timestamp (each representing 30 s), and assay phase. Data from the acclimation phase was not analyzed; analysis in the current study only considered data from the effect phase. Columns 7-8 contain the measured endpoints of time spent and mean distance swam in each zone.

Mirror Response

Juvenile zebrafish from the F1 and F2 generations were placed individually into arenas containing a mirror on one side. The third of the arena closest to the mirror was designated the mirror zone. Fish were allowed to acclimate without external lighting of the arena for 5 min and then swim for 5 min with lighting, enabling fish to see their reflections in the mirror. Time spent and swimming distance were measured to assess zone preference.

The first two columns contain metadata including filename and well location. Each unique combination of filename and location represents an individual fish. Columns 3-6 contain additional metadata, including exposure group (nominal ng/g PFHxA in F0 diet), zone (mirror or full), timestamp (each representing 30 s), and assay phase. Data from the acclimation phase was not analyzed; analysis in the current study only considered data from the effect phase.

Columns 7-8 contain the measured endpoints of time spent and mean distance swam in each zone.
